# Supplementary material for: A thiol‐bound drug reservoir enhances APR‐246‐induced mutant p53 tumor cell death
Source: EMBO Mol Med. 2020 Dec 14;13(2):e10852. doi: 10.15252/emmm.201910852 (PMC7863383; doi:10.15252/emmm.201910852)
Supplement: Supplementary file 1 — Appendix [file EMMM-13-e10852-s001.pdf]

Appendix

A thiol-bound drug reservoir enhances APR-246-induced mutant p53 tumor cell death

Ceder S., Eriksson S.E., Cheteh E.H., Dawar S., Corrales Benitez M., Bykov V.J.N., Fujihara K.M., Grandin M., Li X., Ramm S., Behrenbruch C., Simpson K.J., Hollande F., Abrahmsen L., Clemons N.J. and Wiman K.G.

Table of Contents

Appendix Figures and Legends 3

Appendix Figure S1: Multidrug resistance-associated protein 1 (MRP1) inhibition synergizes with APR-246 3

Appendix Figure S2: MRP1 inhibition potentiates antitumoral activity of APR-246 *in vivo* and *ex vivo* 7

Appendix Figure S3: Inhibition of MRP1 efflux pump activity increases ^{14}C-APR-246/MQ accumulation in cancer cells 9

Appendix Figure S4: MRP1 inhibition leads to intracellular accumulation of GS-MQ and the addition of MQ to glutathione is reversible 11

Appendix Figure S5: APR-246 sensitivity is dictated by the presence of mutant p53, cellular thiol status and drug accumulation 13

Appendix Figure S6: MRP1 inhibitor MK-571 shifts cellular thiol pools, further potentiating APR-246 efficacy 15

Appendix Figure S7: GSH and Cys availability determines APR-246/MQ accumulation and sensitivity to APR-246 17

Appendix Tables 21

Appendix Table S1: IC_{50} values of APR-246 +/- MK-571 and synergy scores 21

Appendix Table S2: Extended information for patient-derived organoids (PDO) 22

Appendix Table S3: Effect of MRP1 multidrug efflux-pump inhibitor MK-571 on ^{14}C-APR-246/MQ accumulation in cancer cells 23

Appendix Table S4: Effect of MRP1, xCT or p53 siRNA knockdown in combination with MRP1 inhibitor MK-571 on ^{14}C-APR-246/MQ accumulation in HCT116 cells 24

Appendix Table S5: Extended information on cells included in the test panel 25

Appendix Table S6: Extended information regarding siRNA included in the test panel 27

Appendix Table Legends 28

Appendix Methods 29

Glutathione reductase (GR) activity assay.. 29

Cellular doxorubicin accumulation assay.. 29

Appendix References 29

Appendix Figure S1 Inhibition of efflux pump MRP1 synergizes with APR-246, related to Figure 1

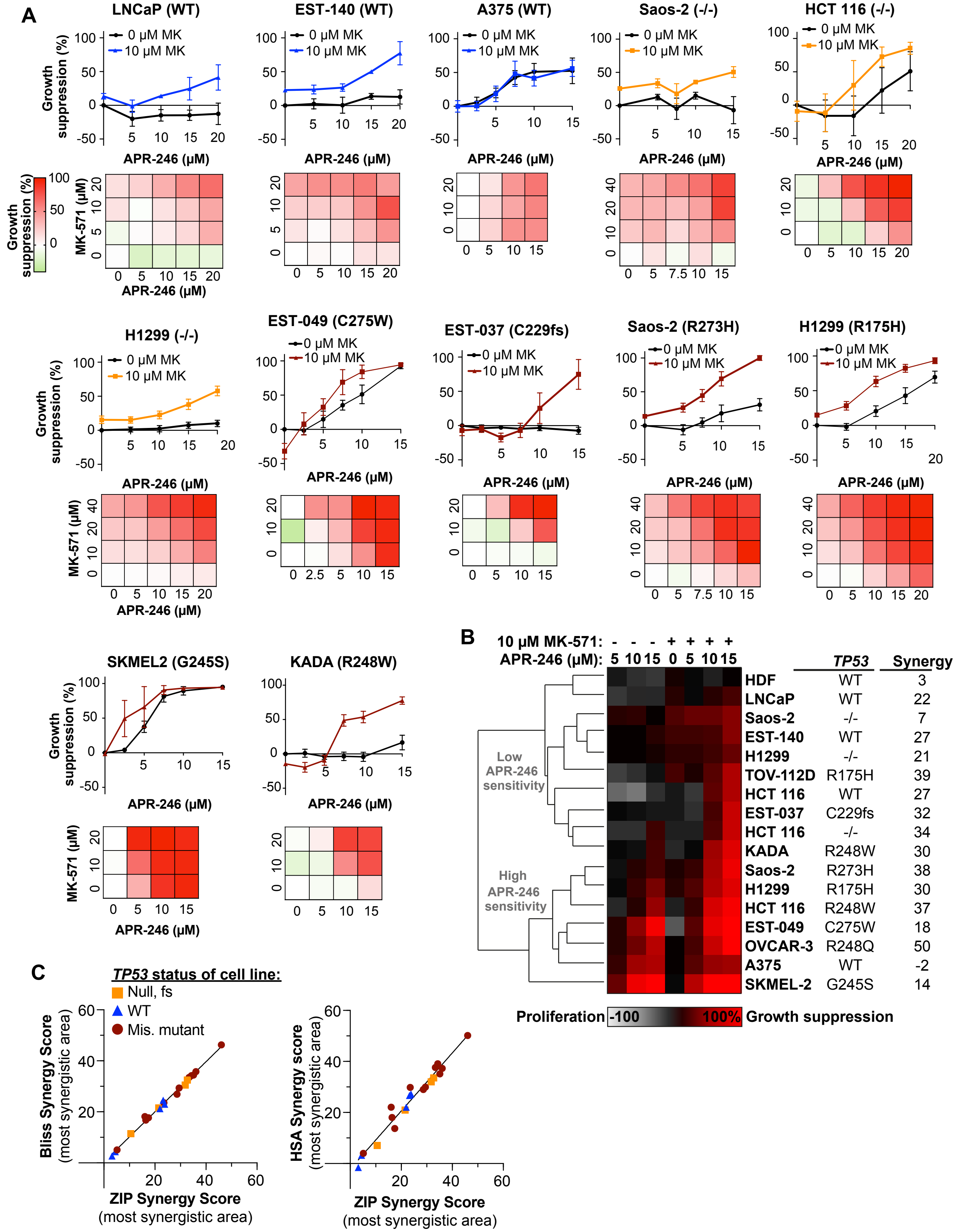

Appendix Figure S1 Inhibition of efflux pump MRP1 synergizes with APR-246, related to Figure 1

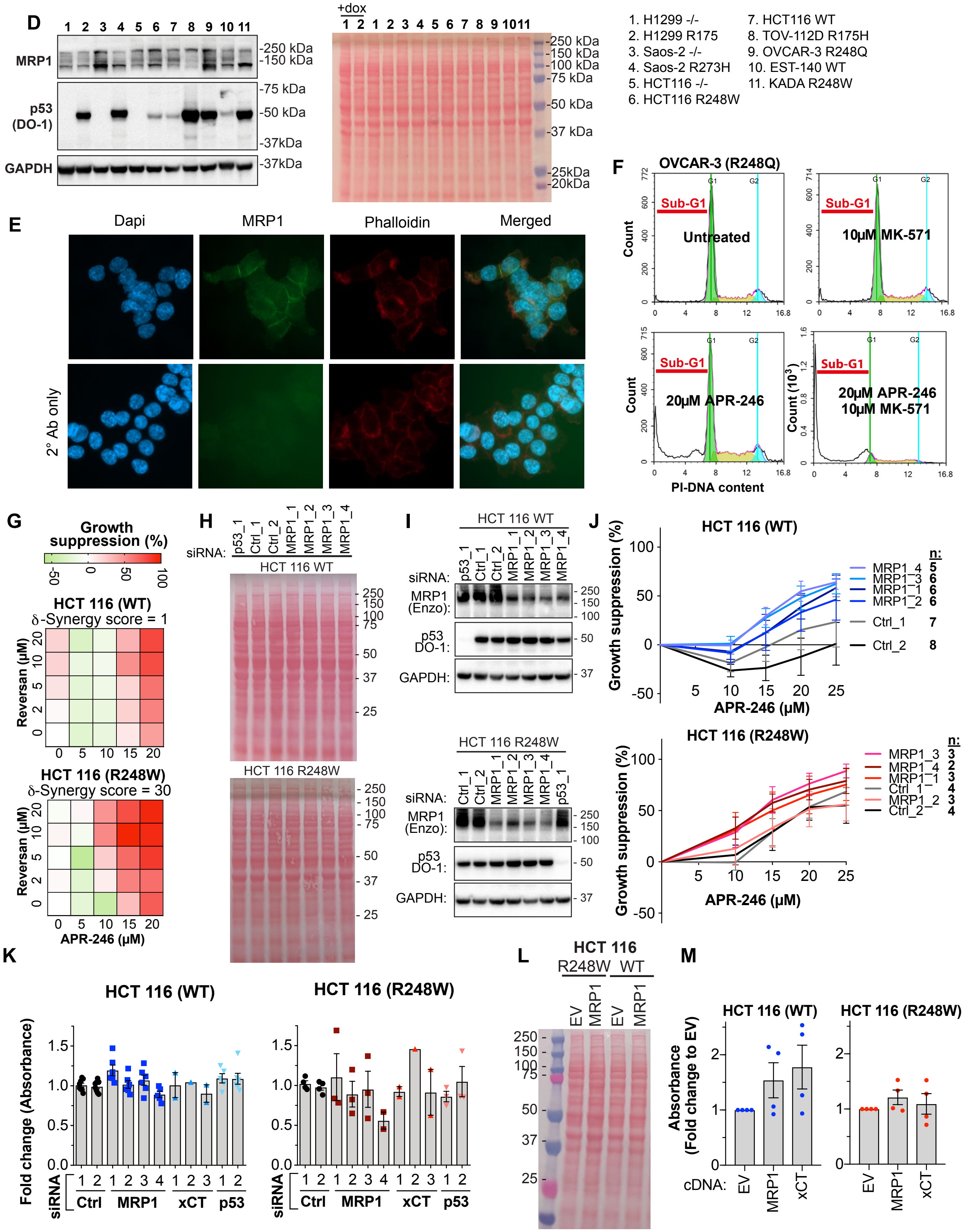

Appendix Figure S1. Multidrug resistance-associated protein 1 (MRP1) inhibition synergizes with APR-246

A. Growth suppression after APR-246 treatment +/- MK-571 for 72h according to the WST-1 assay ($n \geq 3$). See Table S1 for more detailed information including n and IC50 values.
B. Clustering analysis based on growth suppression data (WST-1) from 17 cell lines as shown in Table S1. *TP53* gene status and synergy (δ)-scores according to the ZIP model are indicated ($n \geq 3$). A synergy-score above 0 indicates synergy.
C. Synergy scores of most synergistic area of combination treatment with APR-246 and MK-571. ZIP, Bliss and HSA models were used and plotted against each other. Linear regression HSA vs Bliss R^2= 0.97, p <0.0001, HSA vs ZIP R^2= 0.96, p <0.0001. Detailed information in Table S1.
D. Western blot analysis of p53 (DO-1), MRP1 (Cell Signaling) and GAPDH expression in the indicated cell lines. Ponceau protein staining of the membrane including size marker is shown to the right. Left four lanes of Ponceau stain is the same membrane as Western blot shown in Fig S5A.
E. Immunofluorescence staining showing membrane localization of MRP1 (Cell Signaling) and Phalloidin in HCT116 TP53 R248W cells. 2nd row is the same staining including the secondary antibody (2° Ab) but without the primary MRP1 (Cell Signaling) antibody.
F. Examples of DNA content (determined by PI) flow cytometry histograms of OVCAR-3 cells treated with APR-246 +/- MK-571 at the indicated concentrations. Cells with sub-G1 DNA content (apoptotic cells) are marked. (n=3)
G. Cell growth suppression in HCT116 WT and HCT116 R248W cells as determined by the WST-1 assay after 72h of single or combination treatment with APR-246 +/- MRP1 inhibitor Reversan depicted as heatmaps (n = 3). Synergy (δ)-scores according to the ZIP model are indicated.
H. Ponceau staining and size markers of the Western blot in Fig. 1H.
I. Western blot analysis with another MRP1 antibody (Enzo), p53 (DO-1) and GAPDH of HCT116 WT and R248W cells 48h after transfection with negative control siRNAs or siRNAs targeting MRP1 or p53.
J. Growth suppression after 48h of APR-246 treatment in HCT116 cells transfect with MRP1 siRNA or control siRNA as shown by the WST-1 assay, n in figure. Mean growth suppression with the siRNAs combined is represented in Fig 1I and 7B.
K. Effect of siRNAs against MRP1, xCT or p53 on viability of HCT116 WT and HCT116 R248W cells without any drug treatment as determined by the WST-1 assay. Data is shown as fold change to average cell viability of the two siRNA controls ($n \geq 3$, except xCT_1, xCT_3 where n = 2 and xCT_2 where n= 1, n indicated by dots)
L. Ponceau staining and size markers of western blot membrane in Fig. 1J.

M. Effect of transfected MRP1 and xCT on viability of HCT116 WT and HCT116 R248W cells without any drug treatment as determined by the WST-1 assay. Data is shown as fold change to average cell viability of empty vector transfected cells.

   Data information: Data are represented as mean ± SEM. TP53 status is indicated for each cell line. See Table S6 for extended information on siRNAs.

Appendix Figure S2 MK-571 enhances the anti-tumor activity of APR-246 *in vivo* and *ex vivo*

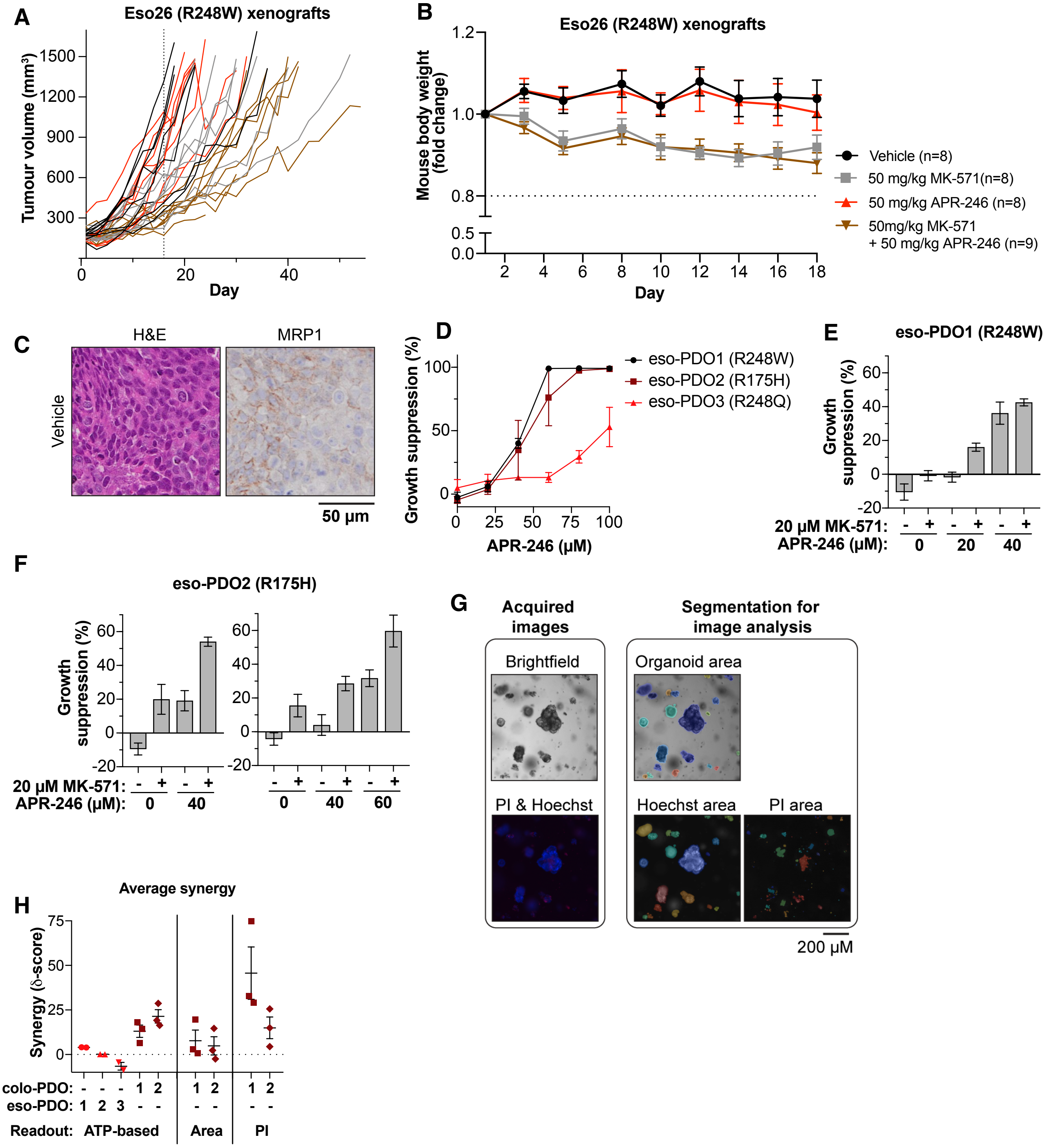

Appendix Figure S2. MRP1 inhibition potentiates antitumoral activity of APR-246 *in vivo* and *ex vivo*

A. Eso26 xenograft tumor volumes of individual mice. Treatments are indicated below the figure.
B. Mean body weight as a percentage change from baseline (pre-treatment weight). Body weight was used as a surrogate marker of toxicity. Ethically acceptable weight loss is defined by the Peter MacCallum Cancer Centre Animal Experimentation Ethics Committee as <20% compared to pre-treatment body weight (above the dotted lines).
C. Representative images of immunohistochemistry staining of MRP1, post treatment/ late timepoint (>22 days after treatment initiation) with APR-246 (50mg/kg) +/- MK-571 (50mg/kg).
D. Growth suppression determined by the ATP-based CTG assay in esophageal cancer patient-derived organoids (eso-PDO) after treatment with APR-246. n = 3.
E. Growth suppression determined by the ATP-based CTG assay in eso-PDO1 after treatment with APR-246 +/- MK-571. Data from one experiment.
F. Growth suppression determined by the ATP-based CTG assay in eso-PDO2 after treatment with APR-246 +/- MK-571. Data from two experiments.
G. Example of segmentation for image analysis. Left: Acquired image from microscope. Right. Cell profiler segmentation used for determining organoid area, Hoechst area and PI area. PI intensity is determined in the PI area segmentation. Organoid area based on brightfield overlaps with Hoechst area.
H. Average synergy score according to the ZIP model based on growth suppression as shown by CTG assay (ATP-based) or image analysis (Area and PI) in eso-PDOs and colo-PDOs. Score above 0 indicates synergy. Each dot indicates n for individual PDOs.

   Data information: Data are represented as mean ± SEM. TP53 status is indicated.

Appendix Figure S3 Inhibition of MRP1 efflux pump activity increases ^{14}C-APR-246/MQ accumulation in cancer cells, related to Figure 1 and 3

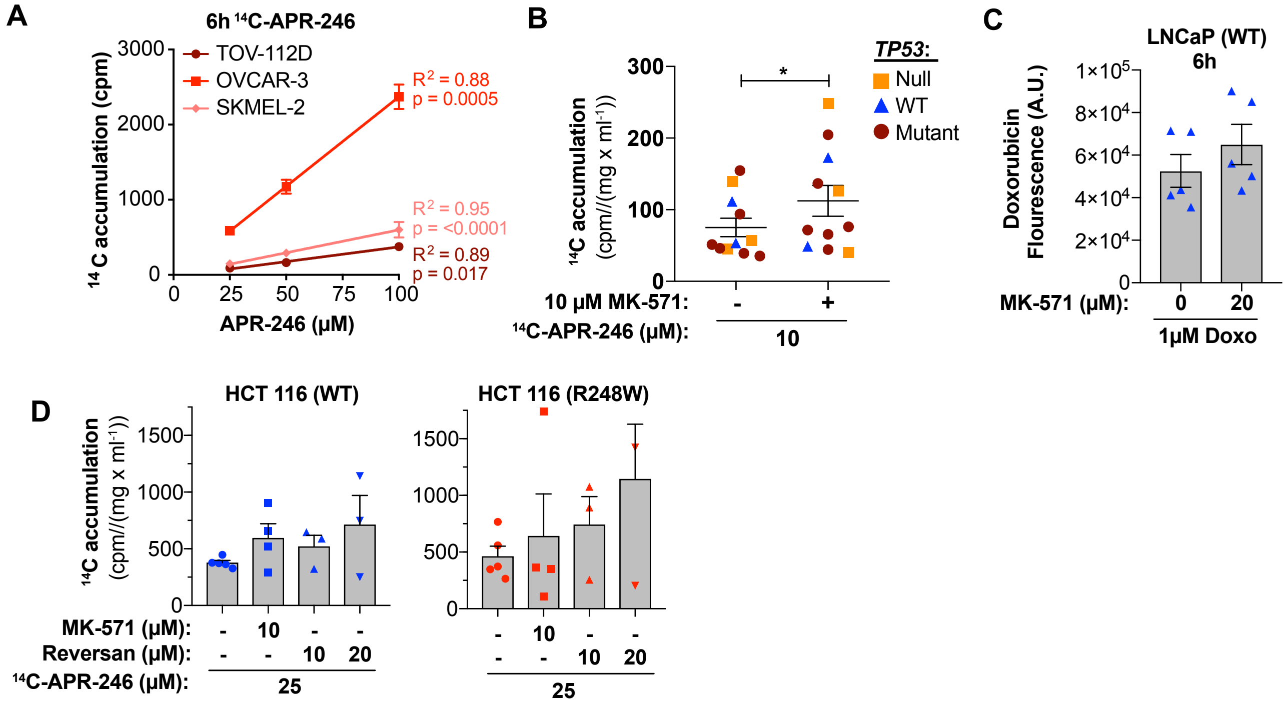

Appendix Figure S3. Inhibition of MRP1 efflux pump activity increases ^{14}C-APR-246/MQ accumulation in cancer cells

A. ^{14}C-accumulation (cpm) after 6h treatment with ^{14}C-APR-246 in mutant *TP53* cell lines TOV-112D (R175H), OVCAR-3 (R248Q) and SKMEL-2 (G245S) (n = 2-3). ^{14}C-APR-246 concentrations were plotted against measured cpm signals and linear regression analysis was conducted.
B. ^{14}C-accumulation (cpm/(mg*ml^{-1})) in 11 cell lines with different *TP53* status at 24h treatment of ^{14}C-APR-246 +/- MK-571 (n≥3). *p=0.02, Wilcoxon matched-pairs signed rank test. Individual cell lines and n are shown in Fig. EV3A and Table S3 .
C. Accumulation of doxorubicin (Doxo) in the presence or absence of MK-571 in LNCaP cells after 6h as assessed by flow cytometry. (n = 5)
D. ^{14}C-accumulation (cpm/(mg*ml^{-1})) in HCT116 WT and HCT116 R248W cells after 24h combination treatment with ^{14}C-APR-246 +/- MK-571 or Reversan (n ≥ 3, n shown by dots).

   Data information: Data are represented as mean ± SEM. TP53 status is indicated for each cell line.

Appendix Figure S4 MRP1 inhibition increases GS-MQ content, forming a reservoir of MQ for interaction with other targets, related to Figure 4

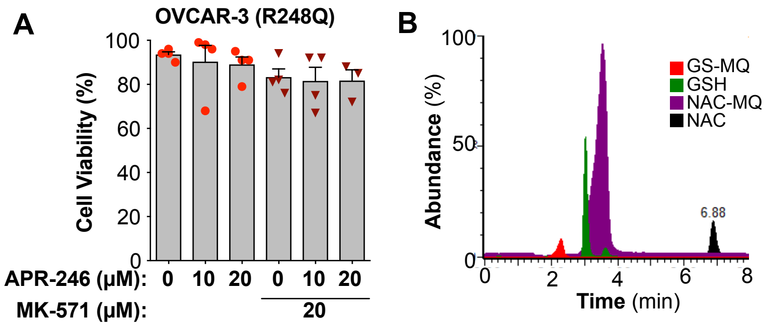

Appendix Figure S4. MRP1 inhibition leads to intracellular accumulation of GS-MQ and the addition of MQ to glutathione is reversible.

A. Viability of OVCAR-3 cells as determined by trypan blue exclusion after 24h treatment with APR-246 +/- MK-571. These cells were used for metabolite analysis by LC-MS.
B. Chromatogram showing retention times for the indicated molecules on LC-MS. *Data information: Indicated values are mean values and error bars are standard error of the mean (SEM).*

Figure Appendix S5 APR-246 sensitivity is dependent on the presence of mutant p53, cellular thiol status and drug accumulation, related to Figure 5

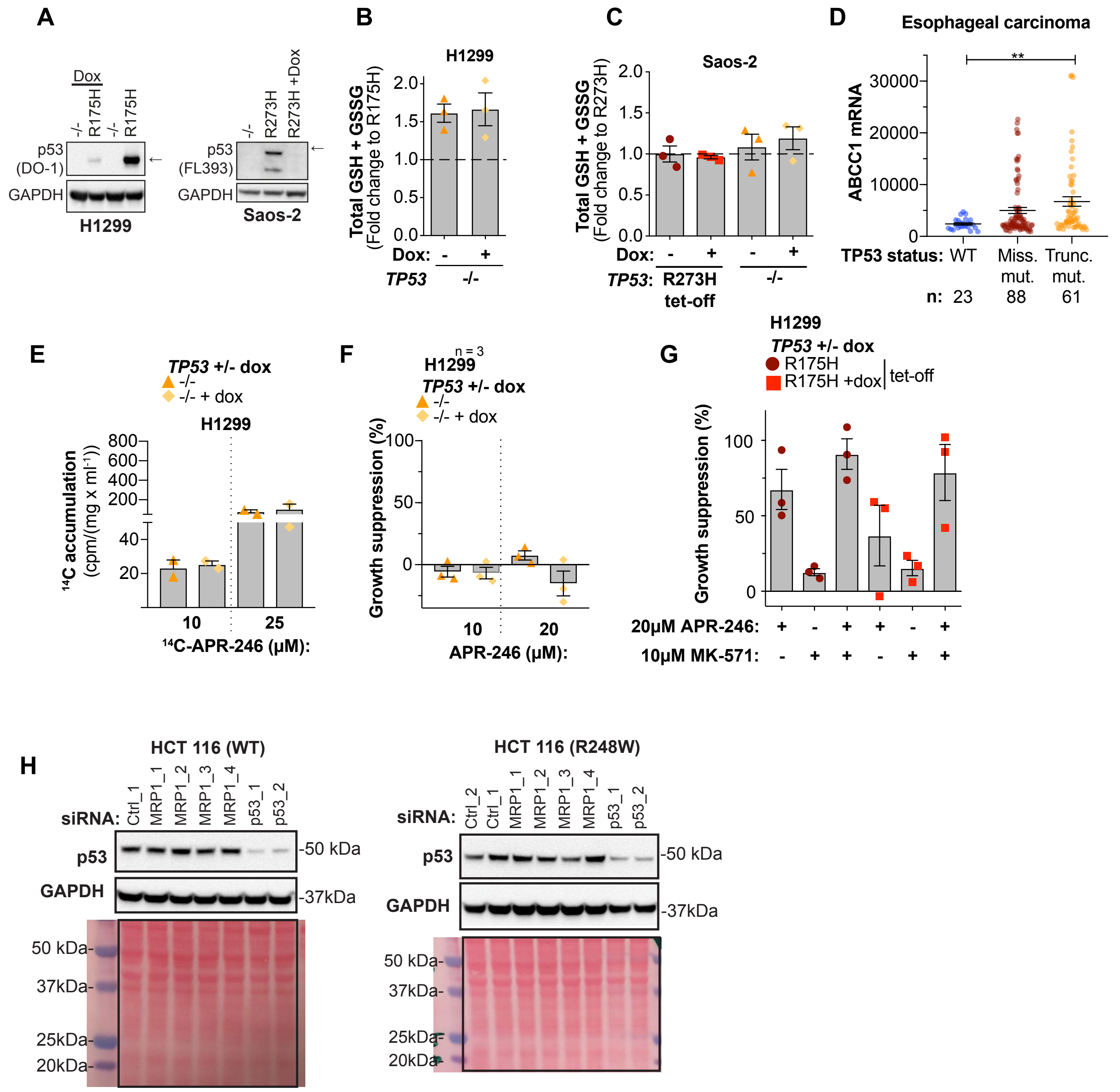

Appendix Figure S5. APR-246 sensitivity is dictated by the presence of mutant p53, cellular thiol status and drug accumulation

A. Western blot of p53 expression in H1299 -/- and R175H cells after 13 days +/- doxycycline (dox), and Saos-2 -/- and R273H (tet-off) cells after 17 days +/- dox. Arrow indicates 50kDa protein marker. The right two lanes of the H1299 blot, i.e. -/- and R175H, are also shown in Fig S1D. Ponceau S staining for the membrane is shown in Fig S1D (four leftmost lanes).
B. Assessment of total GSH+GSSG by a glutathione reductase (GR) re-cycling assay in H1299 -/- cells +/- doxycycline (dox) 24h after seeding (n = 3). The same data for H1299 -/- cells are also shown in Fig 5C. Indicated values are fold change of total glutathione as compared to H1299 R175H cells.
C. Assessment of total GSH+GSSG by a GR re-cycling assay in Saos-2 -/- and R273H cells +/- doxycycline (dox) 24h after seeding (n = 3). Indicated values are fold change of total glutathione as compared Saos-2 R273H cells.
D. mRNA Expression, RSEM (Batch normalized from Illumina HiSeq_RNASeqV2) of *ABCC1* in the TCGA PanCancer atlas of esophageal carcinoma (esca study) (mean and SEM are indicated, *p=0.011) grouped into having no alterations in *TP53* or putative driver mutations (missense or truncating). Statistical analysis by Kruskal-Wallis test and Dunn's multiple comparisons test (indicated p value), n is indicated in the figure.
E. ^{14}C-accumulation (cpm/(mg*ml^{-1})) in H1299 -/- cells +/- dox at 24h treatment with ^{14}C-APR-246 +/- MK-571 (n = 2).The same data for H1299 -/- cells are also shown in Fig 5H.
F. Growth suppression in H1299 -/- cells +/- dox treated with APR-246 for 72h (n = 3) as shown by the WST-1 assay. The same data for H1299 -/- cells are also shown in Fig 5I.
G. Growth suppression in H1299 R175H cells +/- dox treated with APR-246 +/- MK-571 for 72h (n = 3) shown by the WST-1 assay. Part of the data has been shown in Fig 5I.
H. Western blot of p53 expression (DO-1) in HCT116 WT and R248W cells 24h after transfection of siRNA against MRP1 or p53 .
Data information: Indicated values are mean values and error bars are standard error of the mean (SEM). TP53 status is indicated for each cell line.

Appendix Figure S6 MRP1 inhibitor MK-571 shifts cellular thiol pools, further potentiating APR-246 efficacy, related to Figure 6

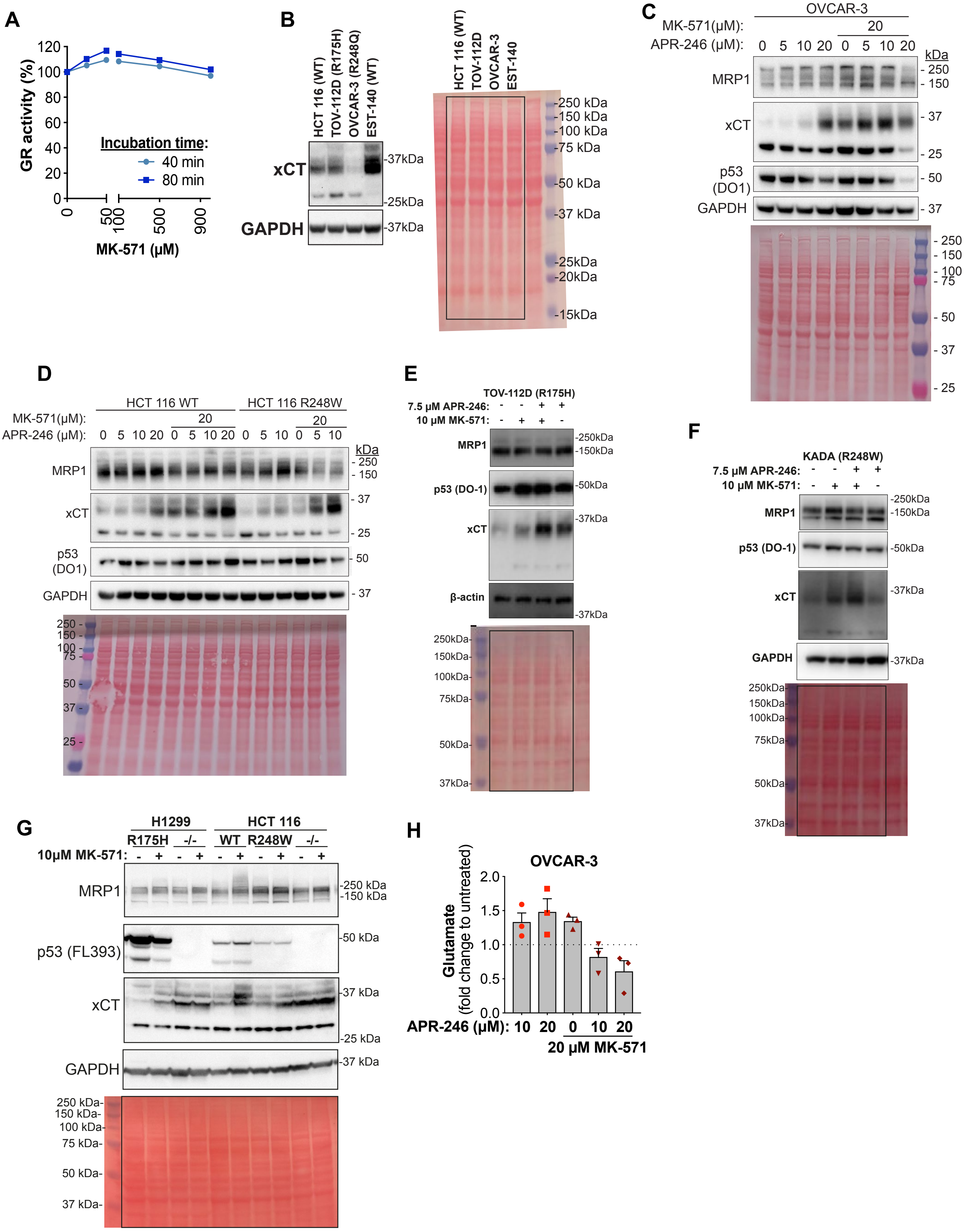

Appendix Figure S6. MRP1 inhibitor MK-571 shifts cellular thiol pools, further potentiating APR-246 efficacy

A. *In vitro* recombinant GR activity assay in the presence of MK-571 at indicated concentrations for 40 or 80 min (n=1).
B. Western blot analysis of xCT and GAPDH and Ponceau S protein loading control of untreated indicated cells. Same membrane is also shown in Fig. S1D.
C. Same Western blot as Fig. 6D and Ponceau S protein loading control for OVCAR-3 cells after 24h of indicated treatments.
D. Same Western blot as Fig. 6E and Ponceau S protein loading control for HCT116 WT and R248W cells after 24h of indicated treatments.
E. Western blot analysis of MRP1 (Cell Signaling), p53 (DO-1), xCT and GAPDH and Ponceau S protein loading control of TOV-112D cells after 24 h of indicated treatments.
F. Western blot analysis of MRP1 (Cell Signaling), p53 (DO-1), xCT and GAPDH and Ponceau S protein loading control of KADA cells after 24 h of indicated treatments.
G. Western blot analysis of MRP1 (Cell Signaling), p53 (FL393), xCT and GAPDH and Ponceau S protein loading control of isogenic H1299 and HCT116 cells with different *TP53* status +/- MK-571 treatment for 24h.
H. Intracellular levels of glutamate in OVCAR-3 R248Q cells as assessed by LC-MS after treatment with APR-246 +/- MK-571 for 24h (n = 3). Indicated values are fold change of glutamate levels to untreated control.
   Data information: Indicated values are mean values and error bars are standard error of the mean (SEM). TP53 status is indicated for each cell line.

Appendix Figure S7 GSH and Cys availibility determines APR-246/MQ accumulation and sensitivity to APR-246, related to Figure 7

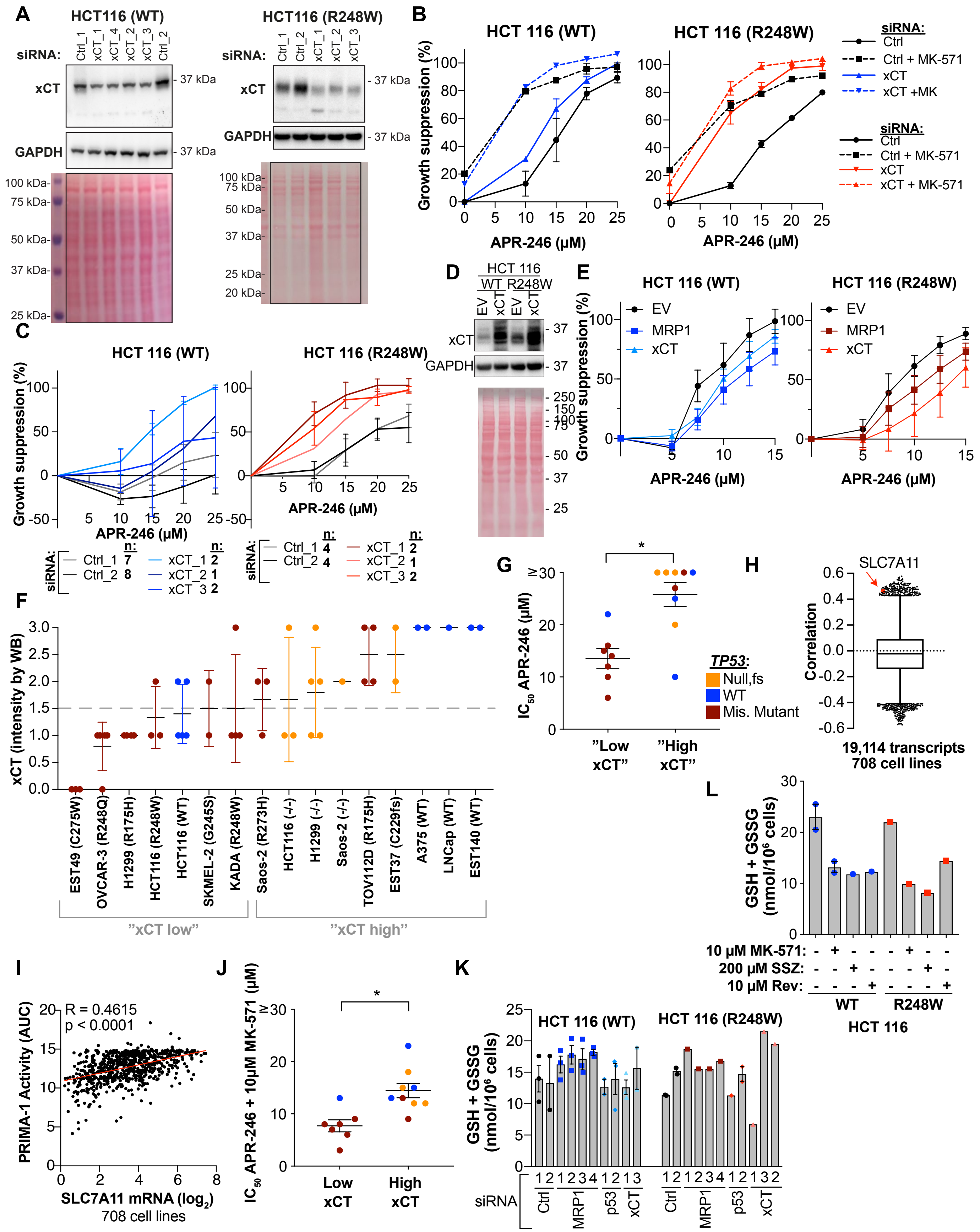

Appendix Figure S7 GSH and Cys availibility determines APR-246/MQ accumulation and sensitivity to APR-246, related to Figure 7

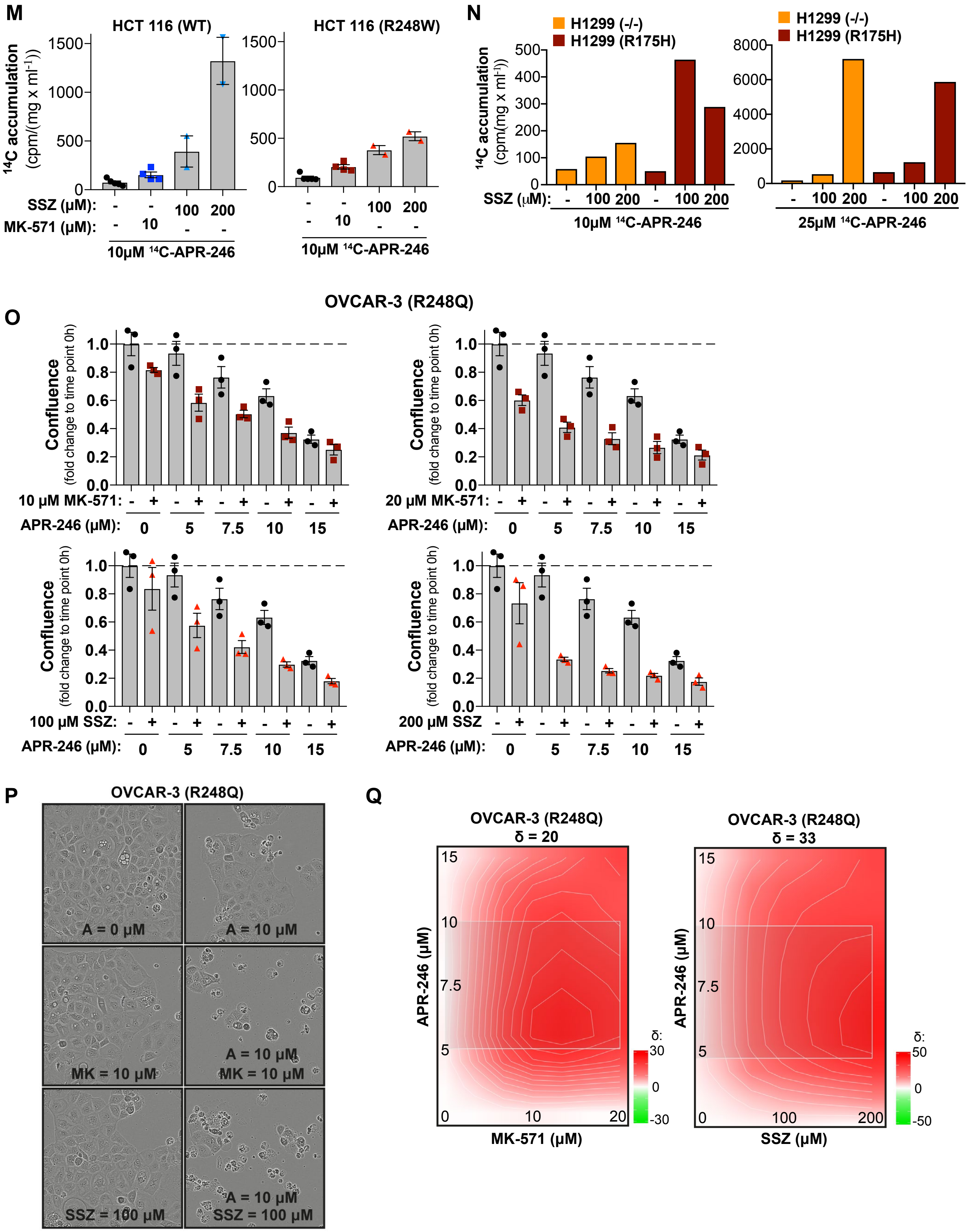

Appendix Figure S7. GSH and Cys availability determines APR-246/MQ accumulation and sensitivity to APR-246

A. Western blot and Ponceau S staining 24h after transient siRNA knockdown of SLC7A11 (xCT) in HCT116 WT or R248W cells.
B. Growth suppression in HCT116 WT and R248W cells after 48h treatment with APR-246 +/- MK-571 and 96h post transfection of siRNA targeting xCT as assessed by the WST-1 assay. Values are means of two different siRNA against xCT (xCT_1 and xCT_2) and two different scrambled control (Ctrl) siRNA (n=1)
C. Growth suppression in HCT116 WT and R248W cells after 48h of APR-246 treatment and 96h post transfection of siRNAs targeting xCT (n=1-2), shown individually, as determined by the WST-1 assay. Two scrambled siRNAs were included as controls (Ctrl) (n ≥ = 7-8) and are the same as shown in Fig S1J ~~S3F~~. Mean growth suppression with the siRNAs combined is represented in Fig 7B.
D. Western blot analysis of xCT and GAPDH in HCT116 WT and R248W cells 72h after transfection with xCT or empty vector (EV).
E. Growth suppression (WST-1 assay) of HCT116 WT and R248W cells transfected with MRP1, xCT or EV after 72h APR-246 treatment (n = 4). Part of data are shown in Fig. 1K.
F. Semi-quantification of xCT protein expression as assessed by Western blotting. Each dot indicates one separate blot. xCT levels were classified as follows: 0= no band (no expression), 1= weak shadow band, 2= band of medium intensity, 3= strong band (equal or similar to GAPDH/beta-actin loading control). Dashed line indicates the middle of the scoring dividing the cell lines into low and high xCT expressing lines.
G. IC_{50} values of APR-246 according to xCT semi-quantified data (Fig S7F) divided into two categories according to xCT expression. *p = 0.005 by Mann Whitney test. IC_{50} values are shown in Table S1.
H. Box-and-whisker plot of Pearson correlations between PRIMA-1 AUC and 19,114 transcripts from the DepMap portal, showing 1st and 99th percentile outlier genes. SLC7A11 is labelled and correlated with lower PRIMA-1 sensitivity.
I. Correlation of PRIMA-1 AUC vs SLC7A11 mRNA in 708 cell lines from the DepMap portal. R and p values were determined by Pearson's correlation
J. IC_{50} values of APR-246 + MK-571 according to xCT semi-quantified data (Fig S7 F) divided into two categories according to xCT expression. *p = 0.003 by unpaired t test. IC_{50} values are shown in Table S1.
K. Total GSH + GSSG in HCT116 WT and R248W cells 48h post transfection of several different siRNAs targeting MRP1 or xCT (SLC7A11) and two scrambled control (Ctrl) siRNA (WT n = 2-3 and R248W n = 1, n indicated by dots) assessed by GR enzyme assay.

L. Total GSH + GSSG in HCT116 WT and R248W cells after 3h of MK-571, Reversan or Sulfasalazine (SSZ) treatment (n = 1, except WT +/- MK-571 n =2) as determined by GR enzyme assay.
M. ^{14}C-accumulation (cpm/(mg*ml^{-1})) in HCT116 WT and R284W cells at 24h of combination treatment with ^{14}C-APR-246 and MK-571 (n=4) or SSZ (n = 2-4).
N. ^{14}C-accumulation (cpm/(mg*ml^{-1})) in H1299 -/- and R175H cells at 24h combination treatment of ^{14}C-APR-246 and Sulfasalazine (SSZ) (n = 1).
O. Cell confluency of OVCAR-3 R248Q cells after 72h of APR-246 +/- MK-571 or SSZ treatment as determined by IncuCyte®. Graph indicates relative values compared to untreated cells (n = 3).
P. Representative area of light microscopy images from IncuCyte® experiments after 72h of APR-246 (A) +/- MK-571(MK) or Sulfasalazine (SSZ) treatment.
Q. Visualization of drug synergy at different compound concentrations (APR-246 +/- MK-571 or +/- SSZ), where red indicates synergy and green indicates antagonistic effect. δ-value (measure of synergy) indicates mean δ-value over the indicated concentration ranges and δ-value above 0 indicates synergy. Plots and δ-values were generated using SynergyFinder.

Data information: Indicated values are mean values and error bars are standard error of the mean (SEM). TP53 status is indicated for each cell line.

Appendix Table S1. IC_{50} values of APR-246 +/- MK-571 and synergy scores, related to Figure 1 and S1

Assay (APR-246 conc. range to determine IC_{50} and synergy)	Cell line (*TP53* status)	n (*)	IC_{50} (µM) APR-246	IC_{50} (µM) APR-246 + 10 µM MK-571	IC_{50} (µM) APR-246 + 20 µM MK-571	Most synergistic area score (up to 20µM MK-571) ZIP	Most synergistic area score (up to 20µM MK-571) Bliss	Most synergistic area score (up to 20µM MK-571) HSA
WST (0-15 or 20µM)	A375 (WT)	5 (4)	10	13	11	3	3	-2
	EST-140 (WT)	3 (1)	≥ 30	15	5	23	25	27
	EST-037 (C229fs)	3	≥ 30	12	6	32	30	32
	EST-049 (C275W)	3	10	6	3	16	17	18
	H1299 (-/-)	7 (6)	≥ 30	18	11	22	22	21
	H1299 (R175H)	7 (5)	16	8	5	24	24	30
	HCT 116 (-/-)	7 (3)	20	12	7	33	32	34
	HCT 116 (R248W)	5 (3)	14	7	4	36	36	37
	HCT 116 (WT)	6 (3)	22	13	8	24	23	27
	HDF (WT)	3	≥ 30	26	17	4	4	3
	KADA (R248W)	4	18	9	8	29	29	30
	LNCaP (WT)	3	≥ 30	23	14	22	21	22
	OVCAR-3 (R248Q)	6 (3)	12	8	3	46	46	50
	Saos-2 (-/-)	5	≥ 30	15	10	11	11	7
	Saos-2 (R273H)	5 (4)	27	9	7	33	33	38
	SKMEL-2 (G245S)	3	6	3	3	17	18	14
	TOV-112D (R175H)	3	≥ 30	13	8	34	34	39
Resazurin (0-100µM)	Eso26 (R248W)	3	31	22	16	16	18	22
	FLO-1 (C277F)	3	35	35	34	5	5	4
	JH-ESoAd1 (G266E)	3	38	27	20	35	34	35
	OACM5.1 (R248Q)	3	30	25	17	29	27	29

* number of experiments for combination treatment with 20 µM MK-571 when **n** is different for this combination treatment.

Appendix Table S2. Extended information for patient derived organoids (PDO), related to Figures 2, S2

PDO ID	Abbreviated as	*TP53* status	Cancer type, site and stage.	Chemotherapy regimen and response	Culturing media
PCC002_ LT1A	**colo-PDO1**	p.H214R	colorectal cancer (rectal primary adenocarcinoma), liver metastasis, TxNxM1.	no treatment, PD	**The basal culture medium (BCM):** Advanced Dulbecco's modified Eagle medium/F12 (12634028 , Life Technologies Australia, Mulgrave, VIC, Australia) supplemented with 200 IU/mL penicillin/streptomycin (15140-122, Life Technologies Australia) and 5 mM Glutamax (35050061, Life Technologies Australia). **For complete culture medium (CCM);** 500 nM A83-01 (SML0788-5MG, Sigma-Aldrich, St Louis, MO, USA), 2x final serum free B-27 Supplement (17504001, Life Technologies Australia), 50 ng/mL human EGF (130-097-749, Miltenyi Biotec, Bergisch Gladbach, Germany), 20 ng/mL Gastrin (G9145-.5MG, Sigma-Aldrich), 10 µM SB202190 (S7067-25mg, Sigma-Aldrich), 1 mM N-Acetyl-L-cysteine (A7250, Sigma-Aldrich) and 10 µM YP-27632 (120129, Abcam, Cambridge, UK) was added into BCM.
yPDB516 _LT1	**colo-PDO2**	p.E224D	colorectal cancer (rectal primary adenocarcinoma), liver metastasis, T3N1bM1.	Pre op FOLFOX, PR	
CAM388	**eso-PDO1**	p.R248W	esophageal adenocarcinoma, primary tumor, T2N0M0.	ECX, TRG4	**Complete media:** AdDMEM/F12 medium supplemented with HEPES (1×,Invitrogen), Glutamax (1×, Invitrogen), penicillin/streptomycin (1×, Invitrogen), B27 (1×, Invitrogen), Primocin (1 mg/ml, InvivoGen), N-acetyl-L-cysteine (1 mM, Sigma) Wnt3a-conditioned medium (50% v/v, L-WNT3A cell line is available from ATCC), RSPO1-conditioned medium (20% v/v, available from Trevigen.), recombinant Noggin protein (0.1 µg/ml, Peprotech), epidermal growth factor (EGF, 50 ng/ml, Peprotech), fibroblast growth factor 10 (FGF10, 100 ng/ml, Peprotech), Nicotinamide (10 mM, Sigma), SB202190 (10 µM, Stem Cell Technologies), and A83-01 (0.5 µM, Tocris).
CAM471	**eso-PDO2**	p.R175H	esophageal adenocarcinoma, primary tumor, T3N3M0.	no treatment	
CAM479	**eso-PDO3**	p.R248Q	esophageal adenocarcinoma, primary tumor, T3N0M0,	ECX, TRG4	

PD: Progressive disease. PR: Partial response. Pre op: Preoperative. FOLFOX: Folinic Acid, Fluorouracil, Oxaliplatin. ECX: Epirubicin, Oxaliplatin, Capetcitabine. Histopathological response described by TRG (tumor regression grade) where 1 indicates complete response and 5 as no regressive changes.

Appendix Table S3. Effect of MRP1 multidrug efflux-pump inhibitor MK-571 on ^{14}C-APR-246/MQ accumulation in cancer cells, related to Figure 3, S3, 7 and S7

	Average ^{14}C accumulation [cpm/(mg × ml-1) ±SEM]							
Cell line	10 µM ^{14}C-APR-246				25 µM ^{14}C-APR-246			
	0 µM MK-571	10 µM MK-571	Fold change (to ^{14}C-APR-246 only)	n	0 µM MK-571	10 µM MK-571	Fold change (to ^{14}C-APR-246 only)	n
**Saos-2 (-/-)**	57 ±33	126 ±71	2.2	3	102 ±26	183 ±28	1.8	3
**Saos-2 (R273H)**	46 ±3	66 ±13	1.4	3	128 ±4	351 ±76	2.7	3
**HCT 116 (WT)**	112 ±38	173 ±30	1.5	5-6	483 ±107	816 ±243	1.7	5-6
**HCT 116 (-/-)**	139 ±28	249 ±70	1.8	4	550 ±92	989 ±324	1.8	4
**HCT 116 (R248W)**	94 ±15	205 ±22	2.2	4-5	462 ±90	642 ±371	1.4	4-5
**H1299 (-/-)**	45 ±11	40 ±6	0.9	5	136 ±31	196 ±27	1.4	5
**H1299 (R175H)**	35 ±8	72 ±15	2.1	4	380 ±98	861 ±216	2.3	4
**TOV-112D (R175H)**	39 ±6	44 ±7	1.1	4	186 ±47	410 ±117	2.2	4
**OVCAR-3 (R248Q)**	155 ±25	137 ±38	0.9	3	330 ±49	475 ±57	1.4	3
**EST-140 (WT)**	54 ±12	49 ±9	0.9	3	112 ±23	173 ±72	1.5	3
**KADA (R248W)**	51 ±7	76 ±13	1.5	3	206 ±54	364 ±159	1.8	3

Appendix Table S4. Effect of MRP1, xCT or p53 siRNA-mediated knockdown in combination with MRP1 inhibitor MK-571 on ^{14}C-APR-246/MQ accumulation in HCT 116 cells, related to Figure 3, S3, 7 and S7

	Average ^{14}C accumulation [cpm/(mg × ml-1) ±SEM]					
siRNA	HCT 116 (WT)			HCT 116 (R248W)		
	0 µM MK-571	10 µM MK-571	n	0 µM MK-571	10 µM MK-571	n
**Ctrl_1**	340 ±88	766 ±109	4	377 ±106	603 ±122	3
**Ctrl_2**	459 ±140	856 ±194	4	408 ±221	735 ±316	3
**MRP1_1**	732 ±283	1471 ±687	4	658 ±374	677 ±266	3
**MRP1_2**	755 ±293	1638 ±607	4	629 ±272	955 ±225	3
**MRP1_3**	754 ±200	1787 ±769	4	986 ±612	907 ±494	3
**MRP1_4**	495 ±184	690 ±87	3	820 ±585	534 ±45	2
**xCT_1**	807 ±106	2527 ±1031	2	937 ±476	1342 ±88	2
**xCT_2**	674	1238	1	589	769	1
**xCT_3**	891 ±393	1865 ±504	2	616 ±167	1819 ±1090	2
**p53_1**	390 ±192	1068 ±347	3	343 ±176	888 ±367	2
**p53_2**	481 ±295	585 ±115	3	372 ±204	550 ±121	2

Appendix Table S5. Extended information on cells included in the test panel, related Figures 1-7, S1-S7

Cell lines	*TP53* status	Origin	Culturing conditions	Source	Comments
**Saos-2 -/-**	-/-	Osteosarcoma	IMDM medium (Hyclone) containing HEPES and L-glutamine, supplemented with 10% FBS (Gibco)	ATCC, ~ 2000	STR profiled (PowerPlex 21) 2017. Not mycoplasma tested.
**Saos-2 R273H**	tet-off his273	Osteosarcoma		Michael Fritsche,Institute for Biomedical Research, Frankfurt, Germany, ~ 2000	Tet off regulated exogenous his273. Not authenticated and mycoplasma tested.
**H1299 -/-**	-/-	Carcinoma, non-small cell lung cancer		Peter Chumakov, Engelhardt Institutet of Molecular Biology, Moscow, Russia, ~ 2000	Purchased from ATCC, Mycoplasma tested (Dapi based) latest 2016. STR profiled (PowerPlex 21) 2017
**H1299 R175H**	tet-off his175	Carcinoma, non-small cell lung cancer			Tet off regulated exogenous his175. Mycoplasma tested (Dapi based) 2014. Not authenticated.
**KADA**	p.R248W	Malignant melanoma		Rolf Kiessling, Karolinska Institutet, Stockholm, Sweden, 2015	Whole exome sequenced by Lars-Gunnar Larsson, Karolinska Institutet, Stockholm, Sweden. Mycoplasma tested (PCR-based) 2017.
**A375**	WT	Malignant melanoma	MEM medium (Gibco), supplemented with 10% FBS, NEAA, pyruvate and L-glutamine (Gibco)	ATCC, 2015	Whole exome sequenced by Lars-Gunnar Larsson, Karolinska Institutet, Stockholm, Sweden. Mycoplasma tested 2015.
**SKMEL-2**	p.G245S	Malignant melanoma		ATCC, 2015	
**EST-037 (ESTDAB-037 / IGR-39)**	p.C229fs	Malignant melanoma	RPMI-1640 medium (HyClone) containing HEPES and L-glutamine, supplemented with 10% FBS	ESTDAB, 2015	
**EST-049 (ESTDAB-049 / Mel-624)**	p.C275W	Malignant melanoma		ESTDAB, 2015	
**EST-140 (ESTDAB-140 / Ma-Mel-42a)**	WT	Malignant melanoma		ESTDAB, 2015	
**HCT 116 WT**	WT	Colorectal carcinoma	McCoys 5a medium (Hyclone) containing L-glutamine, supplemented with 10% FBS	Katarina Johansson/Elias Arnér, Karolinska Institutet, Stockholm, Sweden, passage 4, 2016	Purchased from ATCC. Mycoplasma tested (PCR based) 2019. Not authenticated.
**HCT 116 -/-**	-/-	Colorectal carcinoma		Bert Vogelstein, The Johns Hopkins Medical School and Sidney Kimmel Comprehensive Cancer Center, Baltimore, USA, ~ 2000	Targeted disruption of WT *TP53.* Mycoplasma tested (PCR based) 2019. Not authenticated.
**HCT 116 R248W**	p.R248W/-	Colorectal carcinoma		Bert Vogelstein, The Johns Hopkins Medical School and Sidney Kimmel Comprehensive Cancer Center, Baltimore, USA, ~ 2005	R248W knock-in. Mycoplasma tested (PCR based) 2017. Not authenticated.

Cell lines	*TP53* status	Origin	Culturing conditions	Source	Comments
**OVCAR-3**	p.R248Q	Adenocarcinoma of the ovary	RPMI-1640 medium (HyClone) containing HEPES and L-glutamine, supplemented with 10% FBS and insulin-tranferrin-selenium (Life Technologies)	Aprea Therapeutics AB, passage 4, 2014	Purchased from ATCC. Mycoplasma tested (PCR based) 2017. Not authenticated.
**TOV-112D**	p.R175H	Primary malignant adenocarcinoma of the ovary, endometrioid ovarian carcinoma	DMEM low glucose medium (HyClone) containing L-glutamine, supplemented with 10% FBS	Aprea Therapeutics AB, passage 5, 2015	
**LNCaP**	p.P152P	Prostate carcinoma	RPMI-1640 medium (HyClone) containing L-glutamine, supplemented with 10% FBS	Arne Östman, Karolinska Institutet, Stockholm, Sweden, ~ 2011	Purchased from ATCC. Mycoplasma tested (PCR based) 2014. Not authenticated.
**HDF**	WT	Primary dermal fibroblasts, human	DMEM low-glucose medium (Hyclone), supplemented with 10% FBS	Coriell Institute for Medical Research, AG01523, 2013	Experiments performed during passage 9-18 and passage doubling <34. Mycoplasma tested (Dapi based) 2014.
**OACM5.1**	p.R248Q	Oesophageal adenocarcinoma	RPMI 1640 medium containing 2.5mM L-glutamine (Life Technologies) supplemented with 10% FBS	Rebecca Fitzgerald (University of Cambridge, UK), 2008	STR profiled (PowerPlex 16) and mycoplasma (PCR based) tested 2018
**Eso26**	p.R248W	Oesophageal adenocarcinoma			STR profiled (PowerPlex 16) and mycoplasma (PCR based) tested 2018
**FLO-1**	p.C277F	Oesophageal adenocarcinoma	DMEM containing 2.5mM L-glutamine and 4.5 g/L D-glucose (Life Technologies) supplemented with 10% FBS		STR profiled (PowerPlex 16) and mycoplasma (PCR based) tested 2018
**JH-EsoAd1**	p.G266E	Oesophageal adenocarcinoma	RPMI 1640 medium containing 2.5mM L-glutamine (Life Technologies) supplemented with 10% FBS	James Eshleman (John Hopkins University, MD, USA), 2011	STR profiled (PowerPlex 16) and mycoplasma (PCR based) tested 2018

Appendix Table S6. Extended information regarding siRNA included in the test panel, related to Figure 3 and 7

Name	Gene Target	Product name	Company
**Ctrl_1**	-	AllStars Negative Control 0001027281	Qiagen
**Ctrl_2**	-	siGENOME Non-Targeting siRNA #5 D-001210-05	Dharmacon
**MRP1_1**	ABCC1	Hs_ABCC1_2 FlexiTube siRNA SI00071204	Qiagen
**MRP1_2**	ABCC1	Hs_ABCC1_3 FlexiTube siRNA SI00071211	Qiagen
**MRP1_3**	ABCC1	Hs_ABCC1_4 FlexiTube siRNA SI00071218	Qiagen
**MRP1_4**	ABCC1	Hs_ABCC1_5 FlexiTube siRNA SI03067603	Qiagen
**xCT_1**	SLC7A11	Hs_SLC7A11_2 FlexiTube siRNA SI00104902	Qiagen
**xCT_2**	SLC7A11	Hs_SLC7A11_4 FlexiTube siRNA SI00104916	Qiagen
**xCT_3**	SLC7A11	Hs_SLC7A11_6 FlexiTube siRNA SI02655506	Qiagen
**xCT_4**	SLC7A11	Hs_SLC7A11_3 FlexiTube siRNA SI00104909	Qiagen
**p53_1**	TP53	Hs_TP53_7 FlexiTube siRNA S102623747	Qiagen
**p53_2**	TP53	Hs_TP53_9 FlexiTube siRNA S102655170	Qiagen

Appendix Table Legends

Table S1. IC_{50} values of APR-246 +/- MK-571 and synergy scores

Number of individual experiments (n), IC_{50} (µM) values of APR-246 single treatment and combination treatment with MK-571 and synergy score from 3 different models in indicated cell lines with different *TP53* status. IC_{50} and synergy scores were determined by the WST-1 or Resazurin assays.

Table S2. Extended information for patient-derived organoids (PDO)

Table S3. Effect of MRP1 multidrug efflux-pump inhibitor MK-571 on ^{14}C-APR-246/MQ accumulation in cancer cells

^{14}C-accumulation (cpm/(mg*ml^{-1})) in 11 cell lines with different *TP53* status after 24h treatment with ^{14}C-APR-246 +/- MK-571. Indicated values are mean values and standard error of the mean (SEM). The total number of experiments (n) for each cell line is indicated.

Table S4. Effect of MRP1, xCT or p53 siRNA knockdown in combination with MRP1 inhibitor MK-571 on ^{14}C-APR-246/MQ accumulation in HCT116 cells

^{14}C-accumulation (cpm/(mg*ml^{-1})) in HCT116 *TP53* WT and R248W cells following 24h of ^{14}C-APR-246 treatment +/- MK-571, and 48h after MRP1, xCT or p53 siRNA knockdown. Four, three and two individual siRNA sequences targeting MRP1, xCT and p53 were used, respectively. Two scrambled siRNAs were used as control. Indicated values are mean values and standard error of the mean (SEM). The total number of experiments (n) for each siRNA is indicated.

Table S5. Extended information regarding cell lines in the test panel

Table S6. Extended information regarding siRNAs used in the study

Appendix Methods

Glutathione reductase (GR) activity assay

Glutathione reductase (GR) activity after MK-571 treatment was determined by incubating 4 nM GR, from baker's yeast (S. cerevisiae, G3664 Merck, Germany) in the presence of NADPH and diluted in 0.1 M K_2HPO_4 buffer containing 1 mM EDTA, as described (Carlberg & Mannervik, 1985). Different concentrations of MK-571 were added to the reduced enzyme and samples were incubated at room temperature. Aliquots of samples (10 µl) were transferred to a 96-well plate after 40 and 80 minutes incubation. A mastermix containing GSSG (0.25 mM) and NADPH (0.25 mM) diluted in K_2HPO_4 buffer were added to samples and NADPH-dependent GSSG reduction was followed for 5 minutes at an absorbance of 340 nm using the Versamax microplate reader (Molecular Devices, Switzerland) set at 30 °C.

Cellular doxorubicin accumulation assay

LNCaP cells were seeded and treated with the indicated concentrations of Doxorubicin with or without MK-571. Cells were harvested after 6h treatment. The intracellular doxorubicin accumulation was assessed on 10^4 single events using NovoCyte (Acea Bioscience, USA) flow cytometer and NovoExpress (Acea Bioscience, USA) software. The cells were analyzed with excitation at 488 nm and emission integrated at 530 nm.

Appendix References

Carlberg I, Mannervik B (1985) Glutathione reductase. Methods in enzymology 113: 484-90
