# Supplementary material for: A thiol‐bound drug reservoir enhances APR‐246‐induced mutant p53 tumor cell death
Source: EMBO Mol Med. 2020 Dec 14;13(2):e10852. doi: 10.15252/emmm.201910852 (PMC7863383; doi:10.15252/emmm.201910852)
Supplement: Supplementary file 2 — Expanded View Figures PDF [file EMMM-13-e10852-s002.pdf]

Expanded View Figures

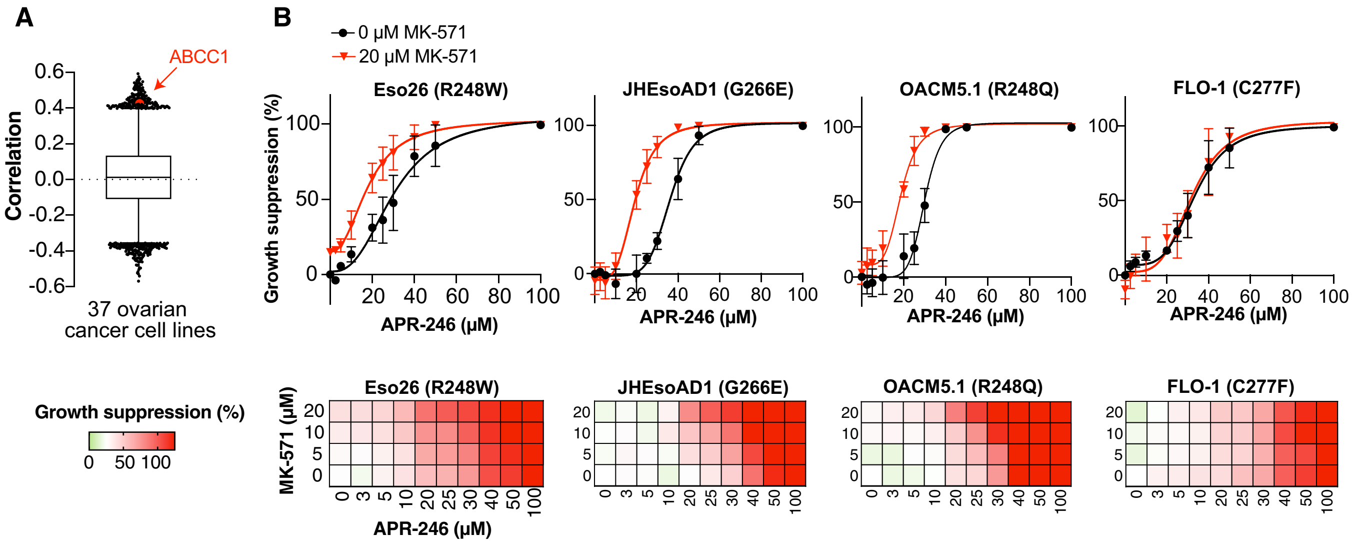

Figure EV1. Multidrug resistance-associated protein 1 (MRP1) inhibition synergizes with APR-246.

A Box-and-whisker plot of Pearson correlations between PRIMA-1 area-under-the-curve (AUC) and 18,827 transcripts from the DepMap portal in 37 ovarian cancer cell lines, central band indicates median, boxes indicate 25th and 75th percentile, and whiskers show 1st and 99th percentile outlier genes. High ABCC1 (MRP1) is labeled and correlates with low PRIMA-1 sensitivity.

B Growth suppression after APR-246 treatment +/− MK-571 for 72 h according to the resazurin assay in esophageal cancer cell lines (n = 3). See Appendix Table S1 for more detailed information. Data are represented as mean ± SEM.

© 2021 The Authors

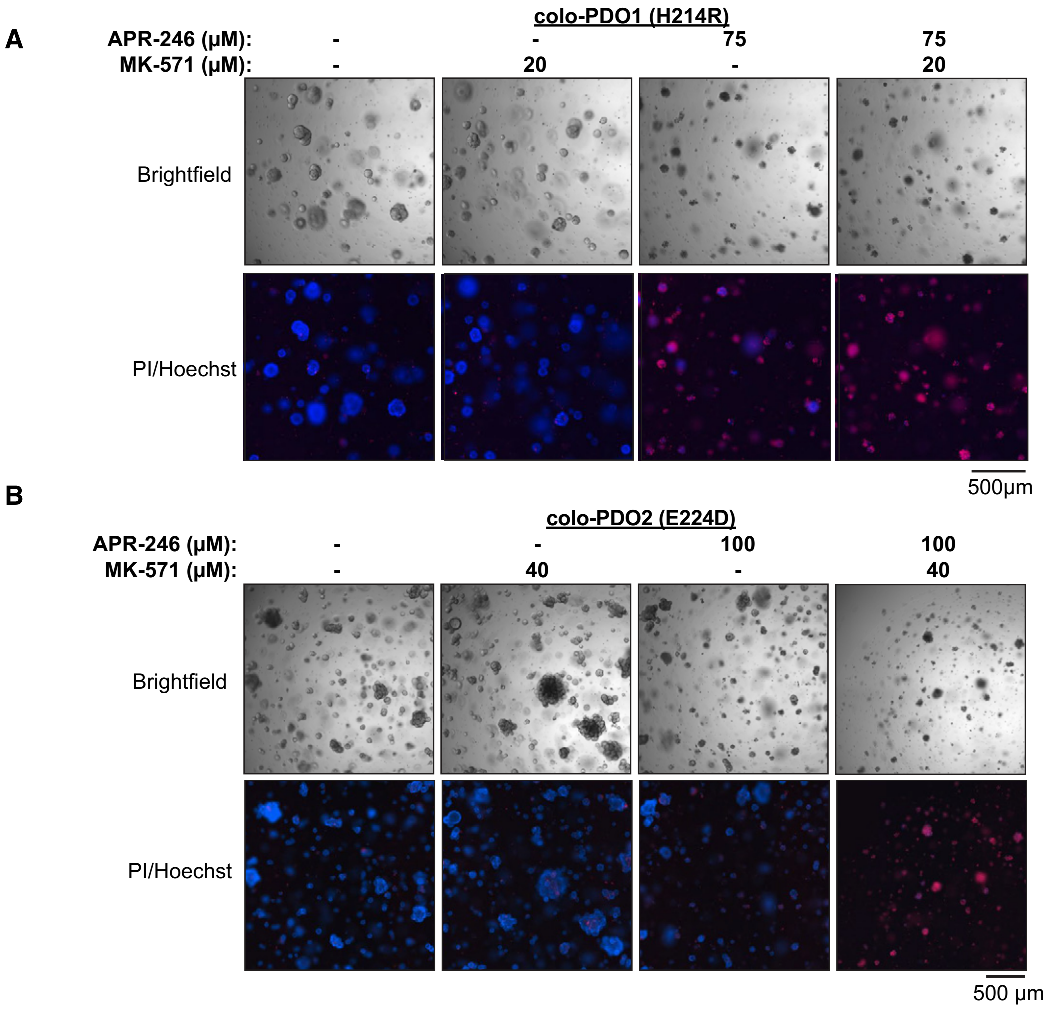

Figure EV2. MRP1 inhibition potentiates anti-tumor activity of APR-246 *in vivo* and *ex vivo*.

A Representative bright-field and PI/Hoechst staining images of colo-PDO1 after 72-h treatment with APR-246 +/− MK-571.

B Representative bright-field and PI/Hoechst staining images of colo-PDO2 after 72-h treatment with APR-246 +/− MK-571.

Data information: *TP53* status is indicated. See Appendix Table S2 for extended information.

Source data are available online for this figure.

© 2021 The Authors

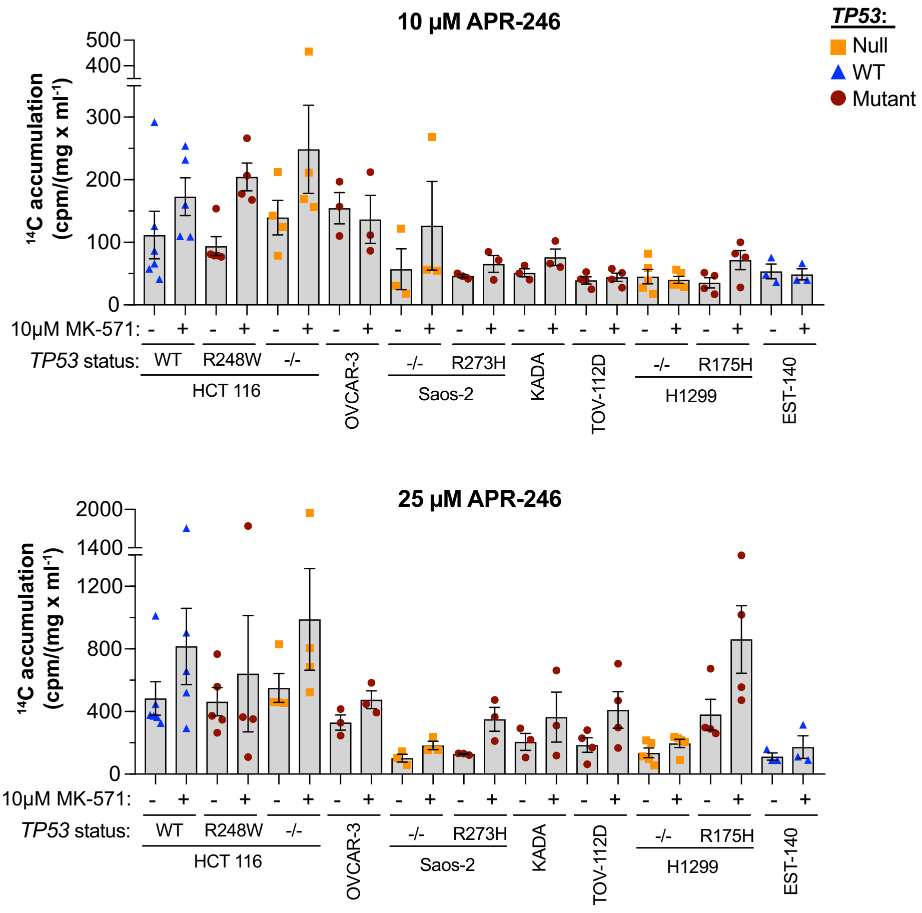

Figure EV3. Inhibition of MRP1 efflux pump activity increases ^{14}C-APR-246/MQ accumulation in cancer cells.

^{14}C accumulation (cpm/mg/ml) in 11 cancer cell lines after 24-h treatment with 10 or 25 μM ^{14}C -APR-246 +/− MK-571 ($n \geq 3$). Detailed information including n is shown in Appendix Table S3. Data are represented as mean ± SEM.

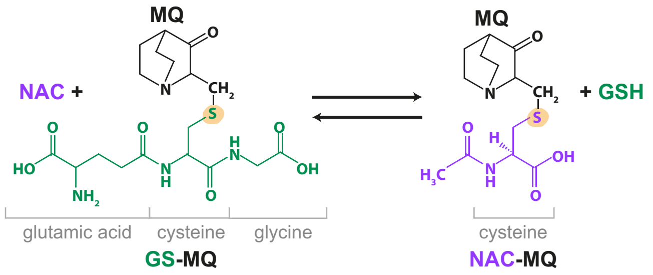

Figure EV4. MQ adduct formation is reversible.

Chemical reaction scheme for the formation of NAC-MQ upon addition of NAC to GSH-MQ conjugates. Yellow indicates reactive thiol.

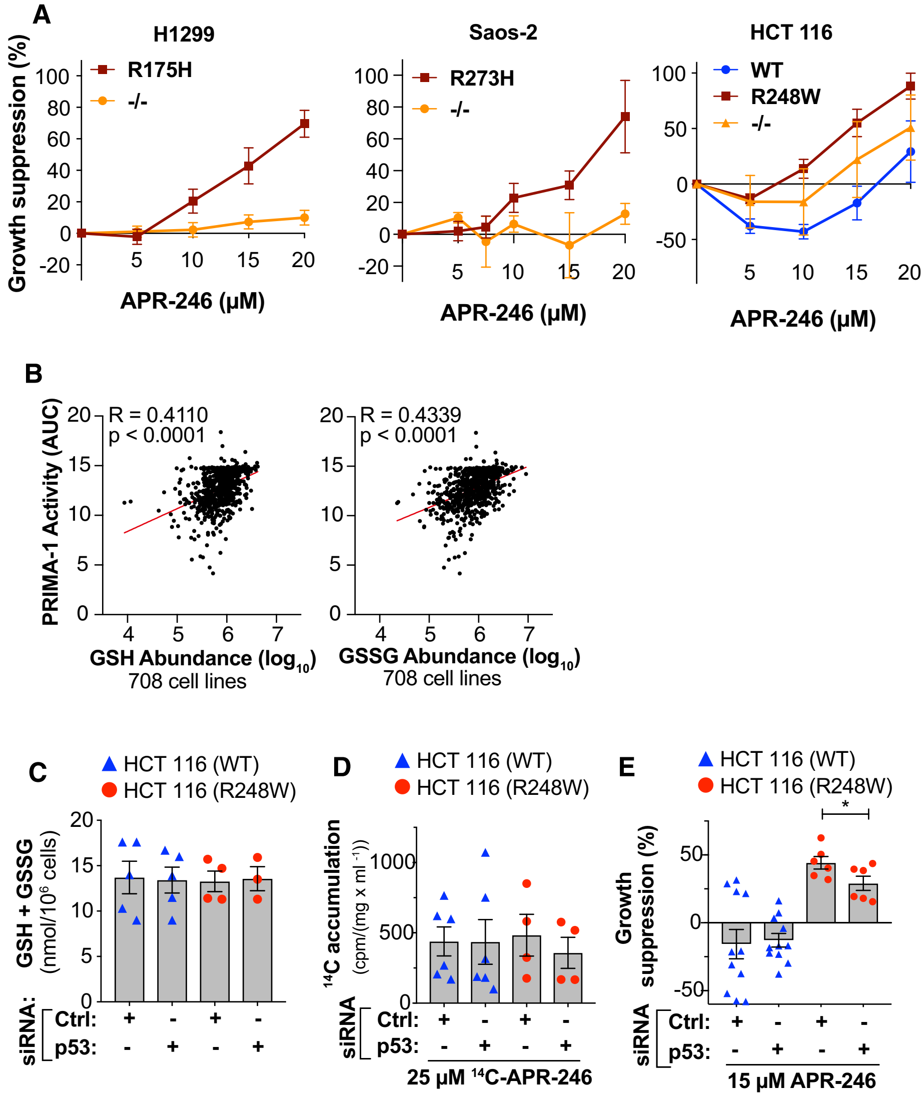

Figure EV5. APR-246 sensitivity is dictated by the presence of mutant p53, cellular thiol status, and drug accumulation.

A Growth suppression of isogenic H1299, Saos-2 and HCT116 cells with different *TP53* status after 72 h APR-246 treatment, as assessed by the WST-1 assay ($n \geq 3$). The data is also shown in Fig 1A and Appendix Fig S1A, *n* in Appendix Table S1.

B Correlation of PRIMA-1 AUC with GSH and GSSG abundance in 708 cell lines from the DepMap portal. *R* and *P* values were determined by Pearson's correlation.

C Total GSH + GSSG by a GR re-cycling assay in HCT116 WT and R248W cells 48 h after siRNA p53 transfection. Values are averages of 1-2 different siRNAs against *TP53* and 1-2 different controls (WT $n = 3$ R248W $n = 2$), individual values are shown in Appendix Fig S7K.

D ^{14}C-accumulation (cpm/(mg/ml)) in HCT116 WT and R248W cells 24 h after treatment with ^{14}C-APR-246 and 48 h after transfection of siRNA against p53. Values are averages of 2 different siRNAs against p53 and 2 different controls (WT $n = 3$ R248W $n = 2$). Values of individual siRNAs are shown in Appendix Table S4.

E Growth suppression in HCT116 WT and R248W cells after 48 h of APR-246 treatment and 96 h post-transfection of +/− siRNAs targeting p53 as determined by the WST-1 assay. Values are averages of 2 different siRNAs against *TP53* and 2 different controls (WT n = 5–6 and R248W $n = 3$) *$P = 0.047$, Paired *t*-test.

Data information: *TP53* status is indicated for each cell line. Data are represented as mean ± SEM.

 © 2021 The Authors

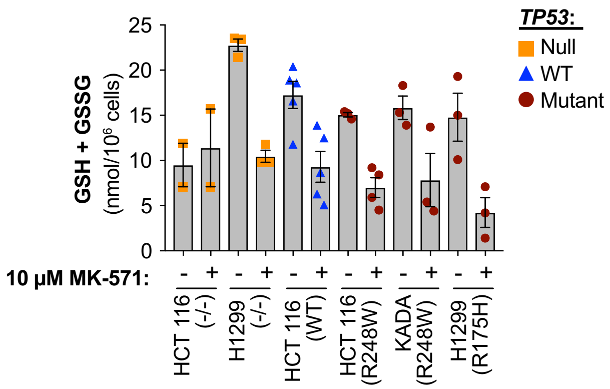

Figure EV6. MRP1 inhibitor MK-571 shifts cellular thiol pools, further potentiating APR-246 efficacy.

Total intracellular glutathione (GSH + GSSG) levels in six cell lines after 24-h incubation +/− MK-571 as determined by a glutathione reductase (GR) re-cycling assay ($n \geq 3$ for each cell line except HCT116 −/− where $n = 2$, n indicates individual experiments). Mean values for each cell line shown in Fig 6A. Data are represented as mean ± SEM.
