# Supplementary figures and images for: A thiol‐bound drug reservoir enhances APR‐246‐induced mutant p53 tumor cell death
Source: EMBO Mol Med. 2020 Dec 14;13(2):e10852. doi: 10.15252/emmm.201910852 (PMC7863383; doi:10.15252/emmm.201910852)

Fig. 1H

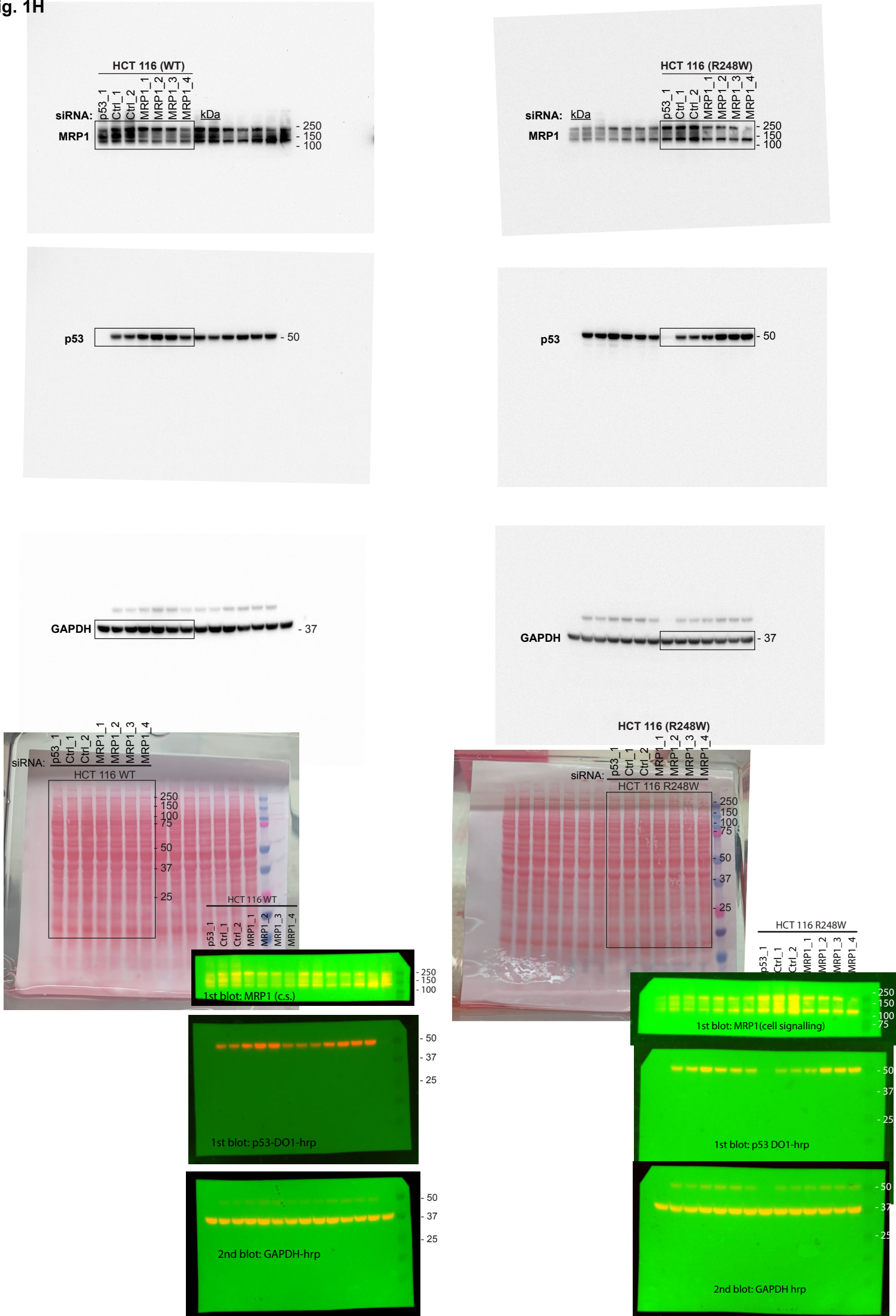

Supplement: Supplementary file 5 — Source Data for Figure 1 [file EMMM-13-e10852-s003.zip › EMM-2019-10852-V4-Figure_1H_Source_Data-sd.pdf]

Fig. 1J

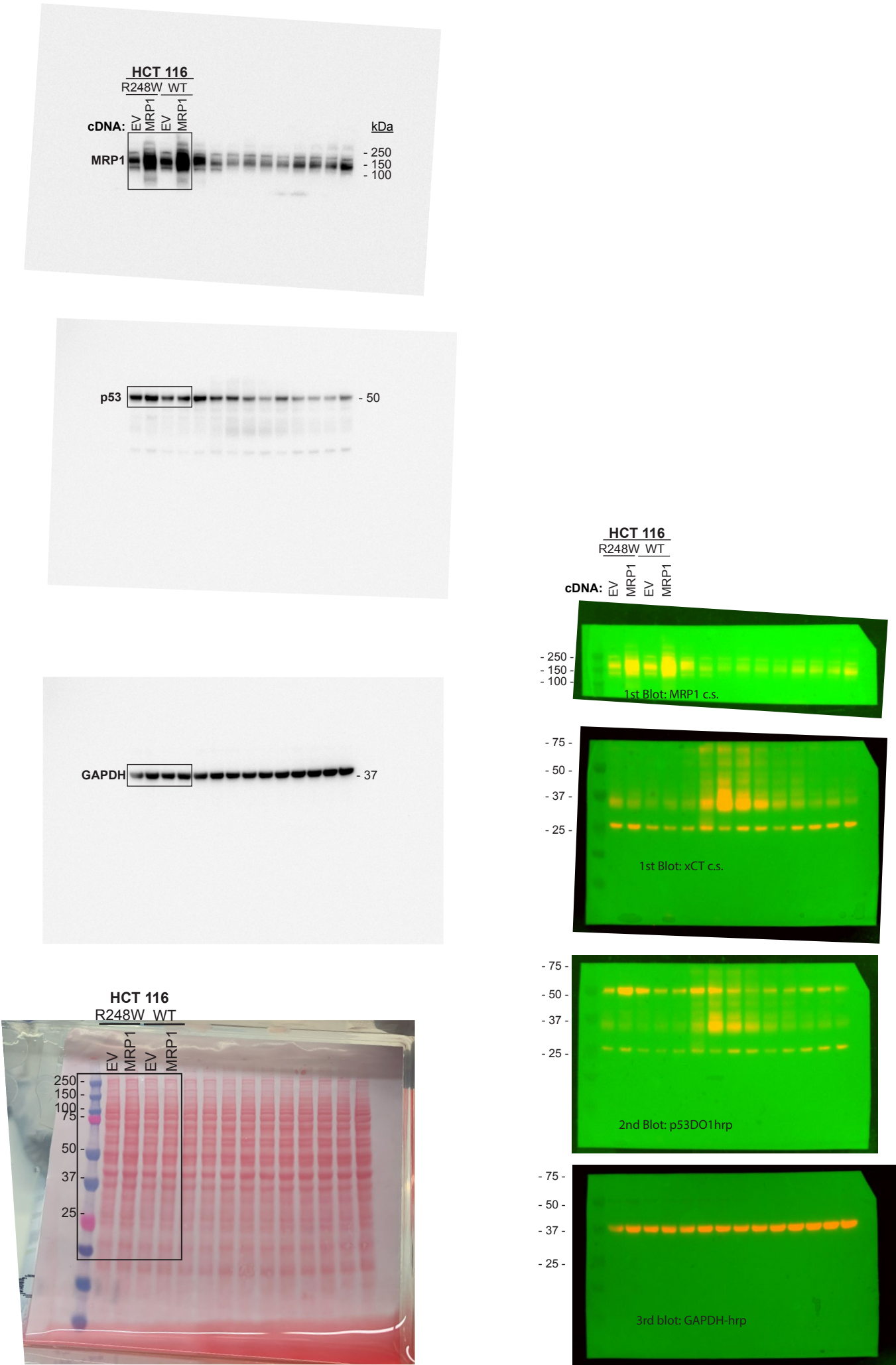

Supplement: Supplementary file 5 — Source Data for Figure 1 [file EMMM-13-e10852-s003.zip › EMM-2019-10852-V4-Figure_1J_Source_Data-sd.pdf]

Fig. 2E

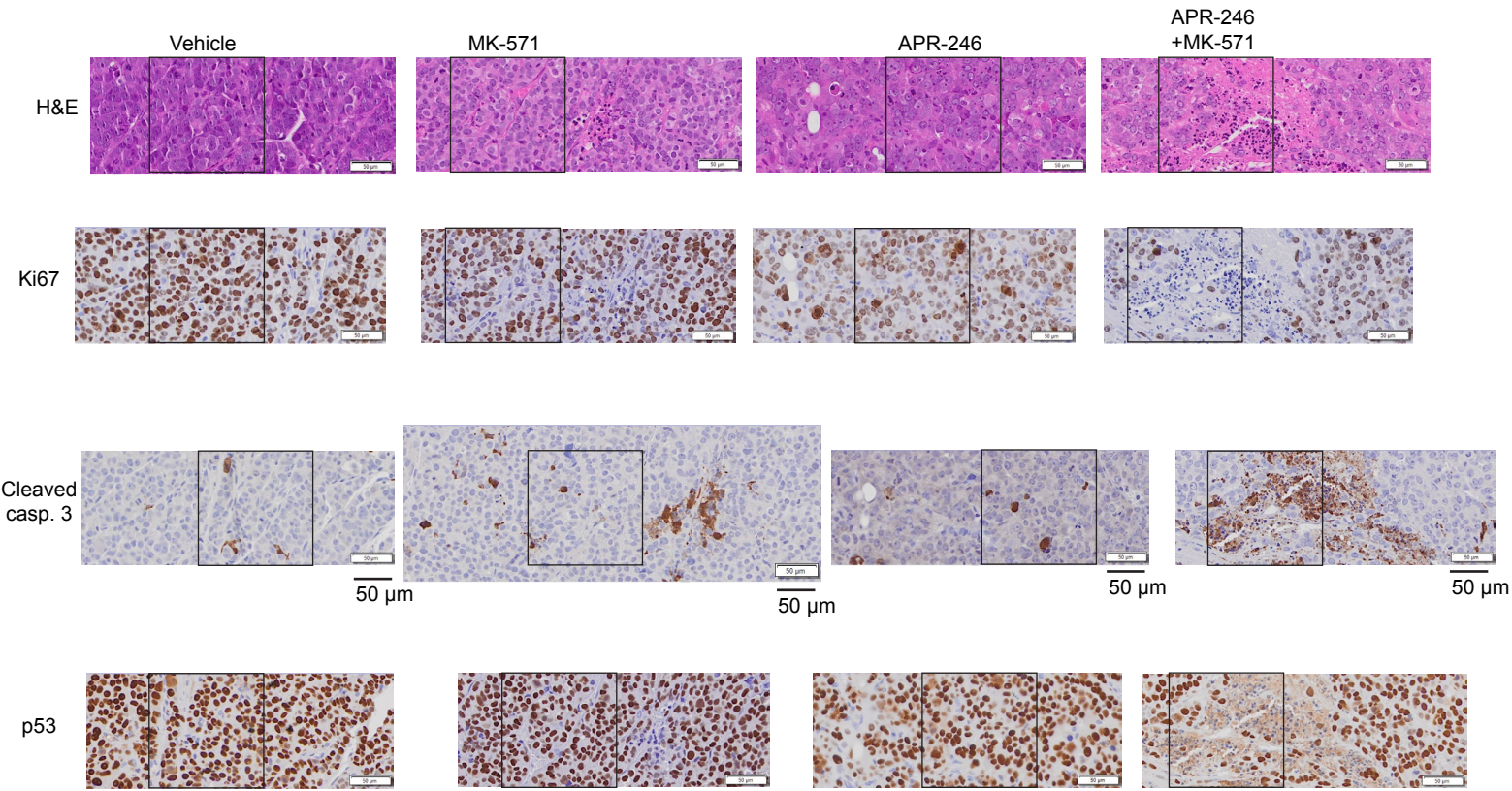

Supplement: Supplementary file 6 — Source Data for Figure 2 [file EMMM-13-e10852-s004.zip › EMM-2019-10852-V4-Figure_2E_Source_Data-sd.pdf]

Fig. 6E and S6D (same western blot)

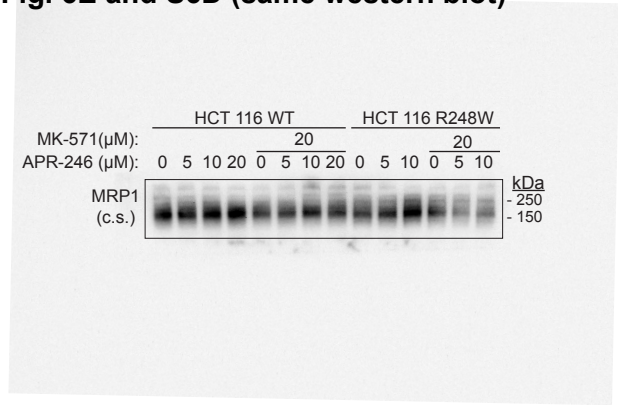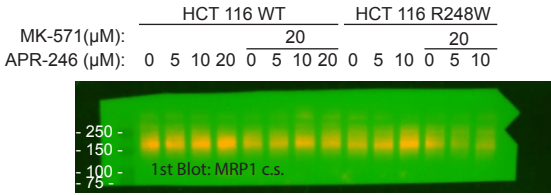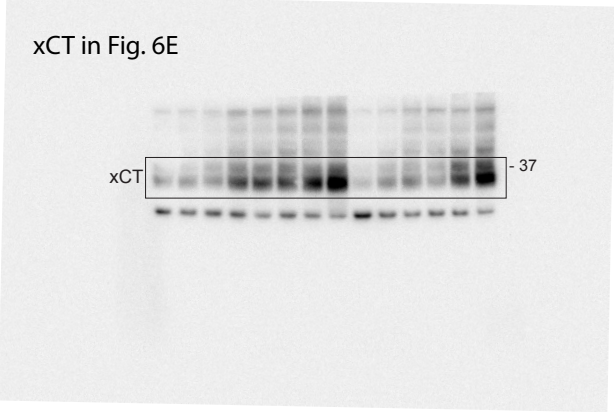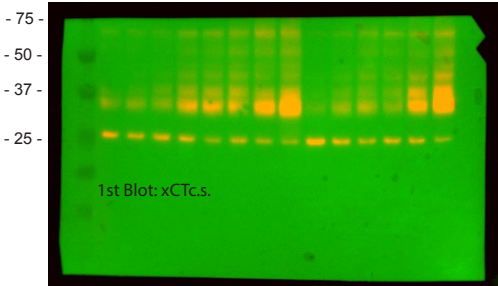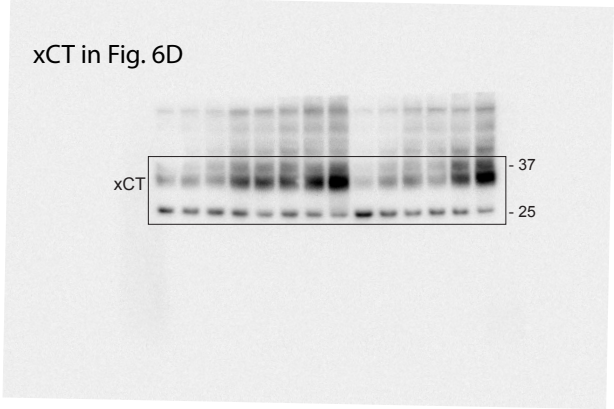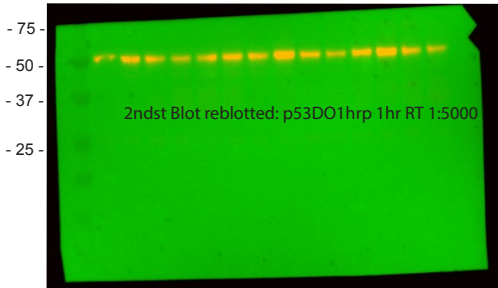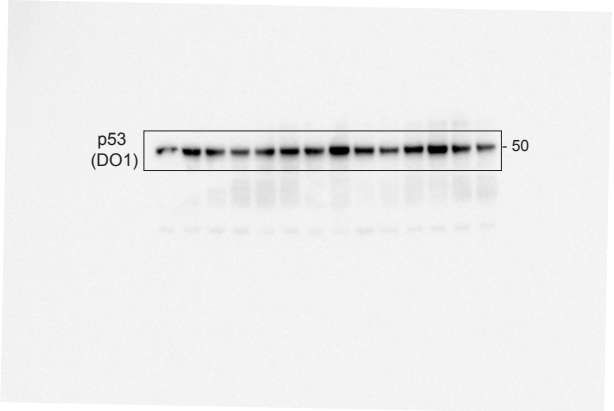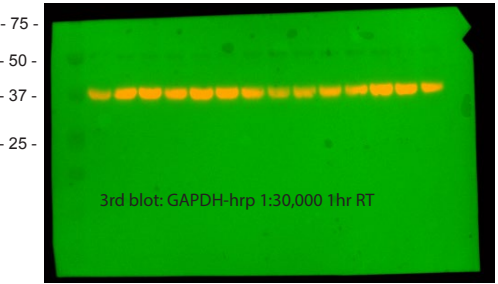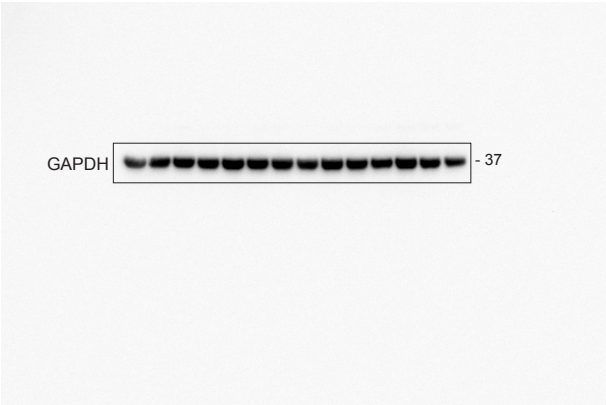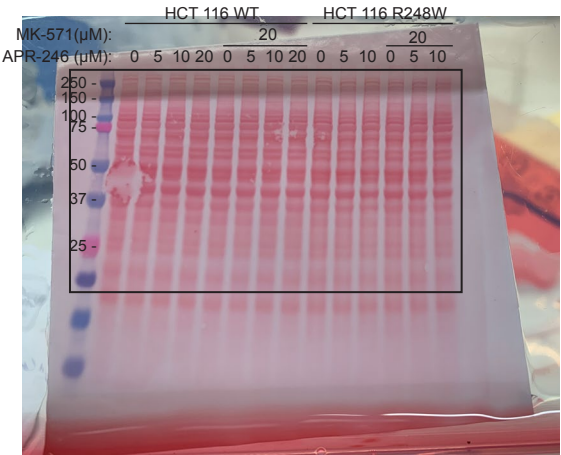

Supplement: Supplementary file 8 — Source Data for Figure 6 [file EMMM-13-e10852-s006.zip › EMM-2019-10852-V4-Figure_6E_and_S6D_Source_Data-sd.pdf]
