# Supplementary material for: A thiol‐bound drug reservoir enhances APR‐246‐induced mutant p53 tumor cell death
Source: EMBO Mol Med. 2020 Dec 14;13(2):e10852. doi: 10.15252/emmm.201910852 (PMC7863383; doi:10.15252/emmm.201910852)

Fig. 6D and S6C (same western)

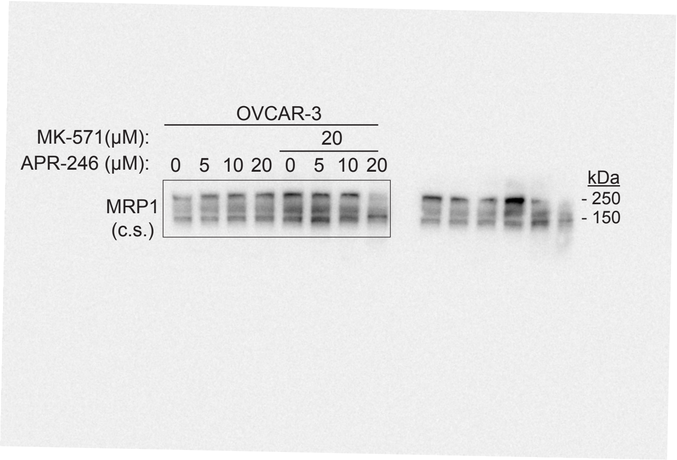

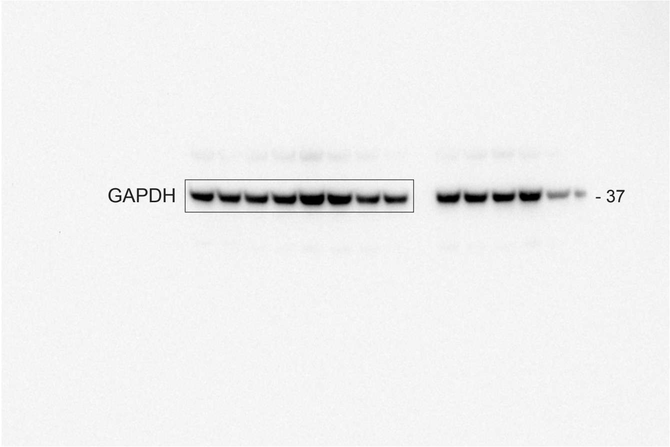

xCT in Fig 6D:

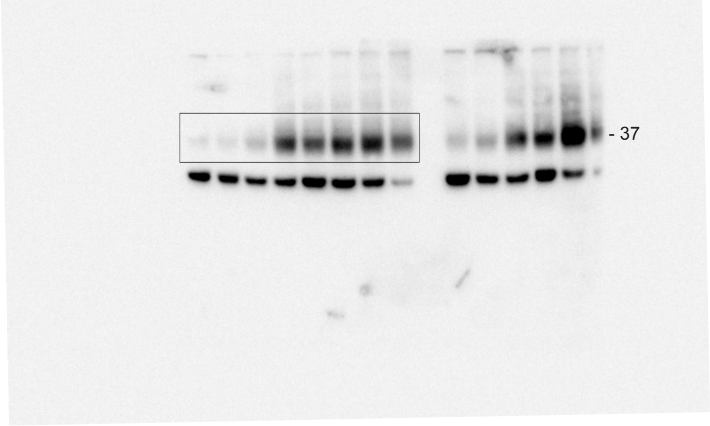

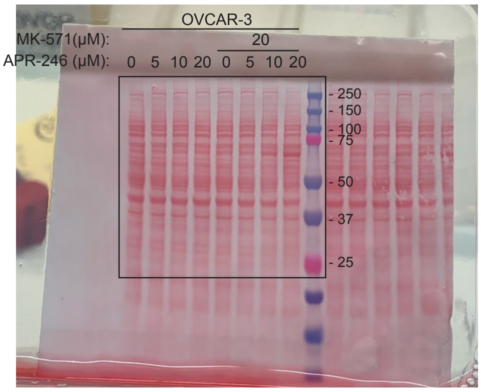

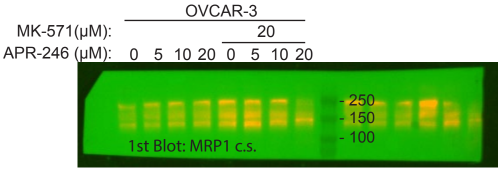

xCT in Fig S6C:

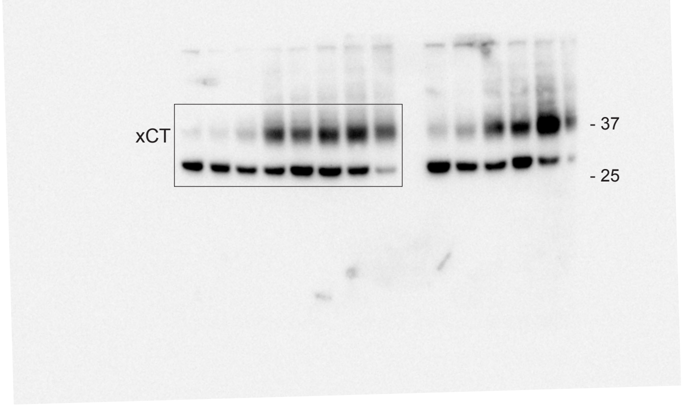

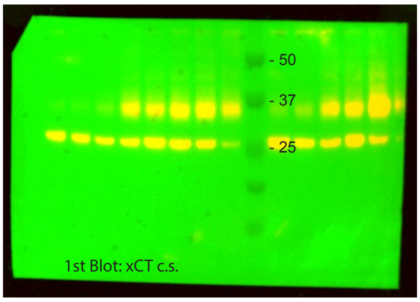

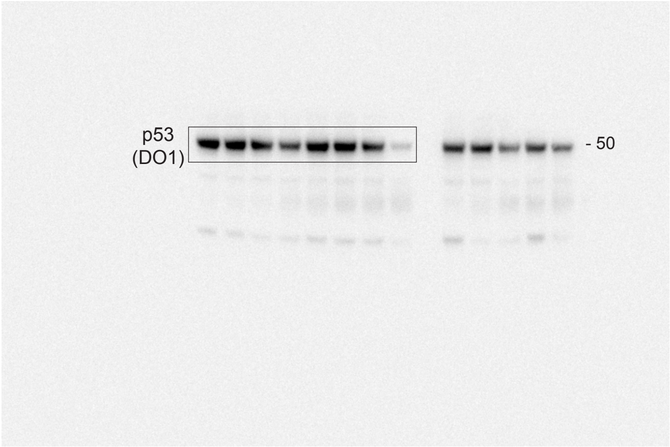

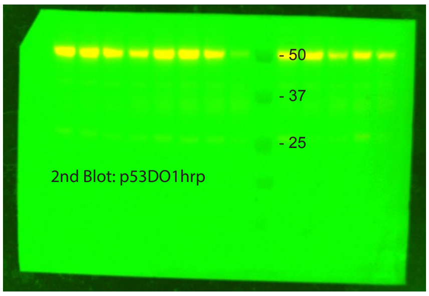

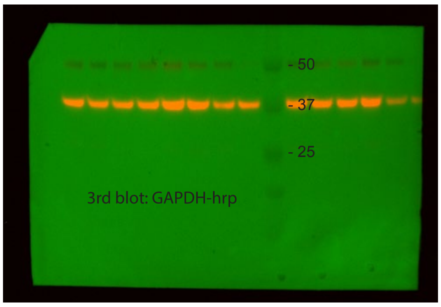

Supplement: Supplementary file 8 — Source Data for Figure 6 [file EMMM-13-e10852-s006.zip › EMM-2019-10852-V4-Figure_6D_and_S6C_Source_Data-sd.pdf]
