# Supplementary material for: A thiol‐bound drug reservoir enhances APR‐246‐induced mutant p53 tumor cell death
Source: EMBO Mol Med. 2020 Dec 14;13(2):e10852. doi: 10.15252/emmm.201910852 (PMC7863383; doi:10.15252/emmm.201910852)
Supplement: Supplementary file 3 — Source Data for Expanded View and Appendix [file EMMM-13-e10852-s007.zip › EV Figure Source Data.zip!/Figure EV2B.pdf]

Fig. EV2B

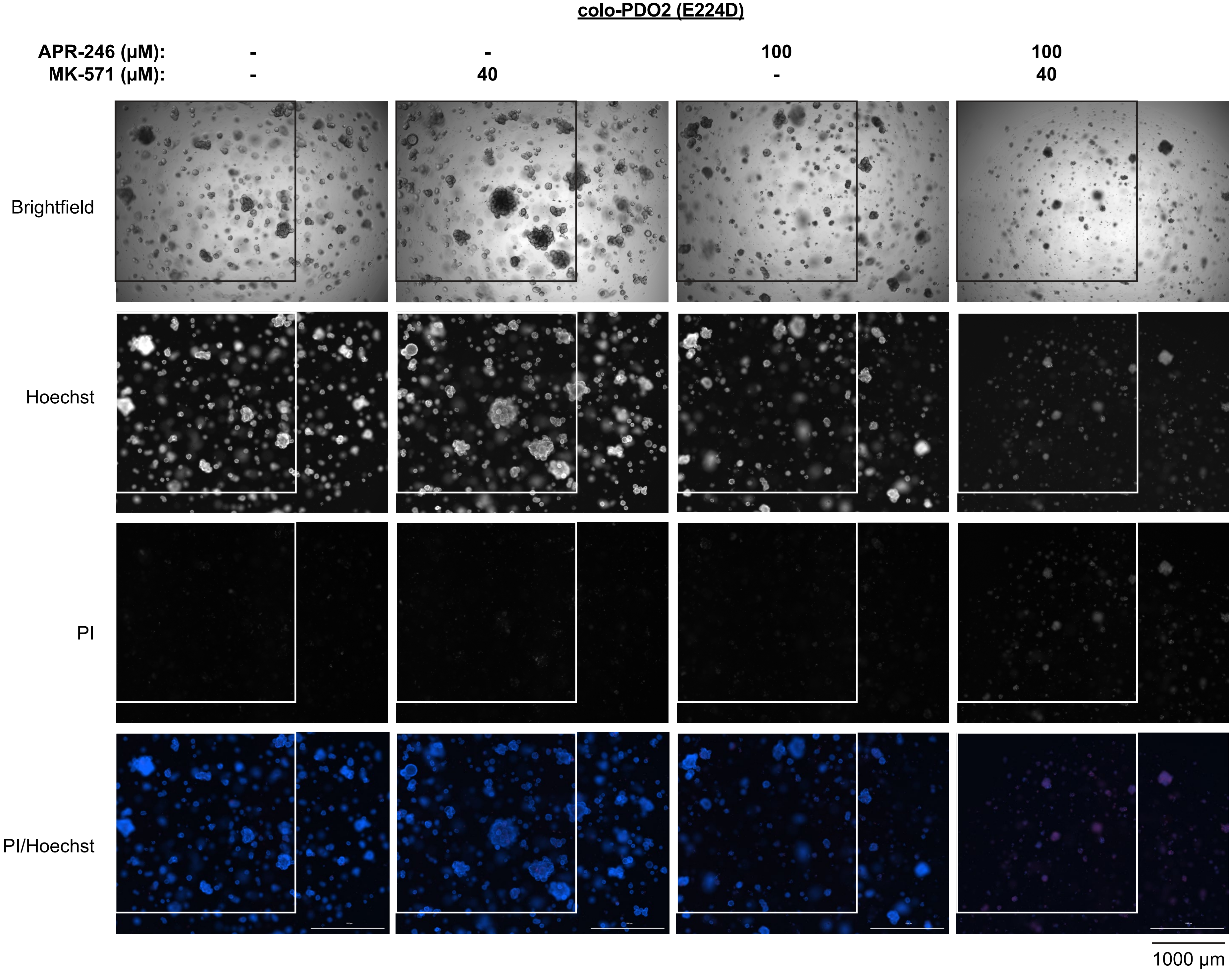
colo-PDO2 (E224D)
APR-246 (µM): - - 100 100
MK-571 (µM): - 40 - 40
Brightfield
Hoechst
PI
PI/Hoechst
1000 µm
