# Supplementary material for: A thiol‐bound drug reservoir enhances APR‐246‐induced mutant p53 tumor cell death
Source: EMBO Mol Med. 2020 Dec 14;13(2):e10852. doi: 10.15252/emmm.201910852 (PMC7863383; doi:10.15252/emmm.201910852)
Supplement: Supplementary file 3 — Source Data for Expanded View and Appendix [file EMMM-13-e10852-s007.zip › Appendix Figure Source Data.zip!/Fig S5.zip!/Figure S5H.pdf]

Fig. S5H

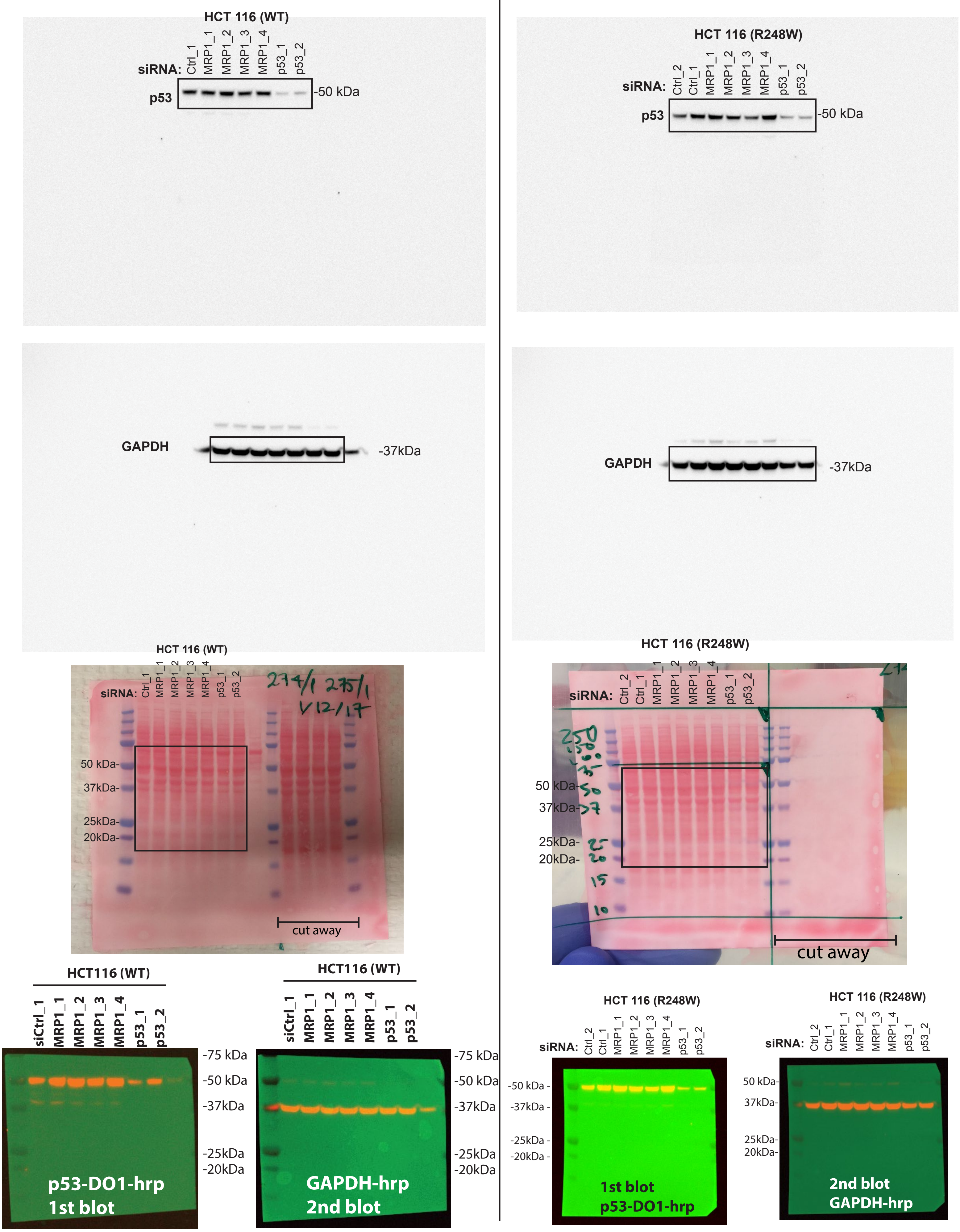
HCT 116 (WT)
siRNA: Ctrl_1 MRP1_1 MRP1_2 MRP1_3 MRP1_4 p53_1 p53_2
p53
-50 kDa
HCT 116 (R248W)
siRNA: Ctrl_2 Ctrl_1 MRP1_1 MRP1_2 MRP1_3 MRP1_4 p53_1 p53_2
p53
-50 kDa
GAPDH
-37kDa
GAPDH
-37kDa
HCT 116 (WT)
siRNA: Ctrl_1 MRP1_1 MRP1_2 MRP1_3 MRP1_4 p53_1 p53_2
50 kDa-
37kDa-
25kDa-
20kDa-
cut away
HCT 116 (R248W)
siRNA: Ctrl_2 Ctrl_1 MRP1_1 MRP1_2 MRP1_3 MRP1_4 p53_1 p53_2
50 kDa-
37kDa-
25kDa-
20kDa-
cut away
HCT116 (WT)
siCtrl_1 MRP1_1 MRP1_2 MRP1_3 MRP1_4 p53_1 p53_2
-75 kDa
-50 kDa
-37kDa
-25kDa
-20kDa
p53-DO1-hrp
1st blot
HCT116 (WT)
siCtrl_1 MRP1_1 MRP1_2 MRP1_3 MRP1_4 p53_1 p53_2
-75 kDa
-50 kDa
-37kDa
-25kDa
-20kDa
GAPDH-hrp
2nd blot
HCT 116 (R248W)
siRNA: Ctrl_2 Ctrl_1 MRP1_1 MRP1_2 MRP1_3 MRP1_4 p53_1 p53_2
-50 kDa -
-37kDa -
-25kDa -
-20kDa -
1st blot
p53-DO1-hrp
HCT 116 (R248W)
siRNA: Ctrl_2 Ctrl_1 MRP1_1 MRP1_2 MRP1_3 MRP1_4 p53_1 p53_2
50 kDa-
37kDa-
25kDa-
20kDa-
2nd blot
GAPDH-hrp
