# Supplementary material for: A thiol‐bound drug reservoir enhances APR‐246‐induced mutant p53 tumor cell death
Source: EMBO Mol Med. 2020 Dec 14;13(2):e10852. doi: 10.15252/emmm.201910852 (PMC7863383; doi:10.15252/emmm.201910852)

Fig. S1D and S5A (same western blot)

1. H1299 -/-
2. H1299 R175
3. Saos-2 -/-
4. Saos-2 R273H
5. HCT116 -/-
6. HCT116 R248W
7. HCT116 WT
8. TOV-112D R175H
9. OVCAR-3 R248Q
10. EST-140 WT
11. KADA R248W

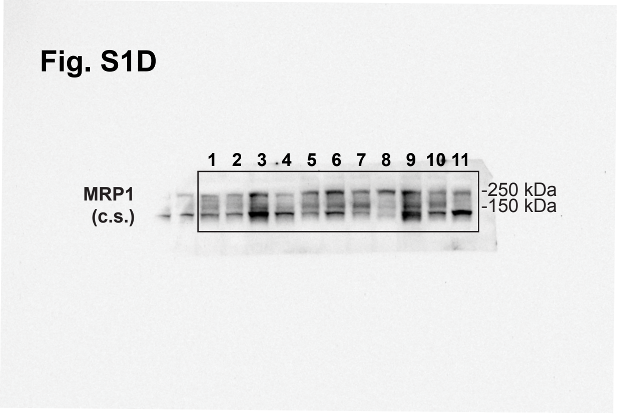

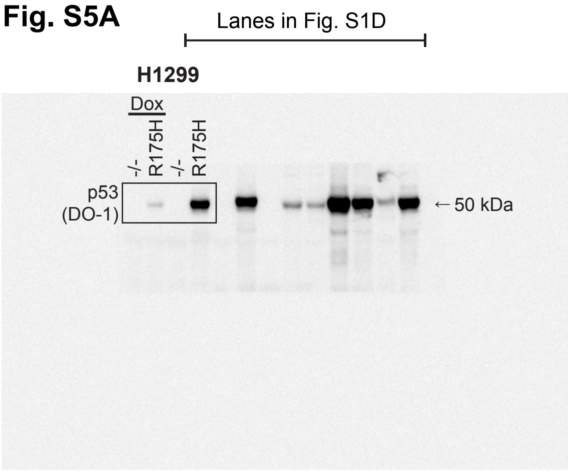

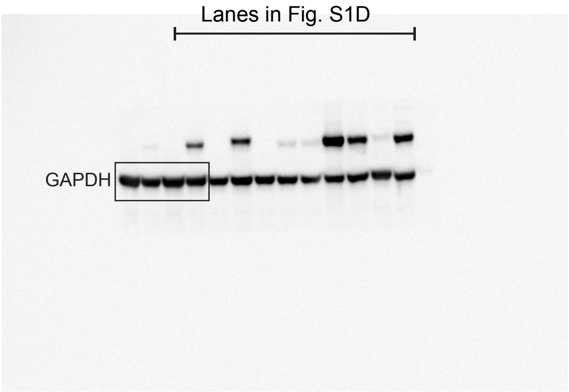

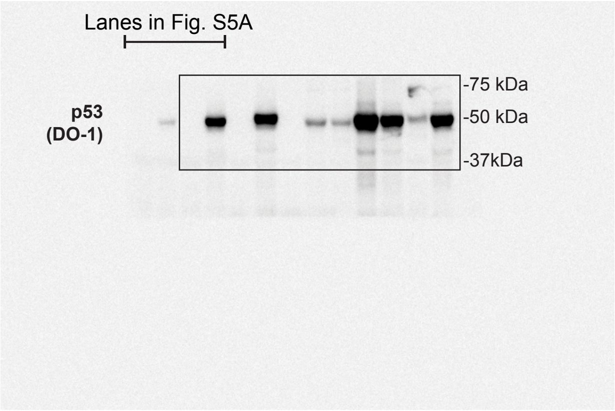

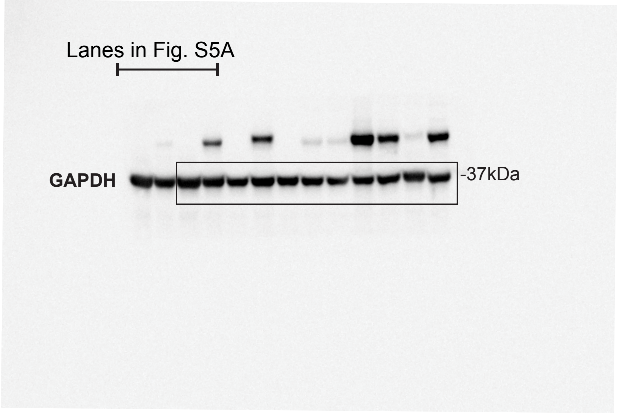

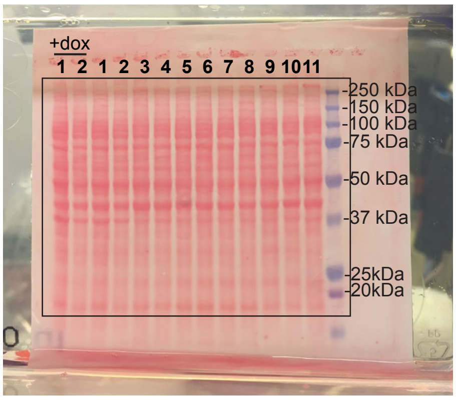

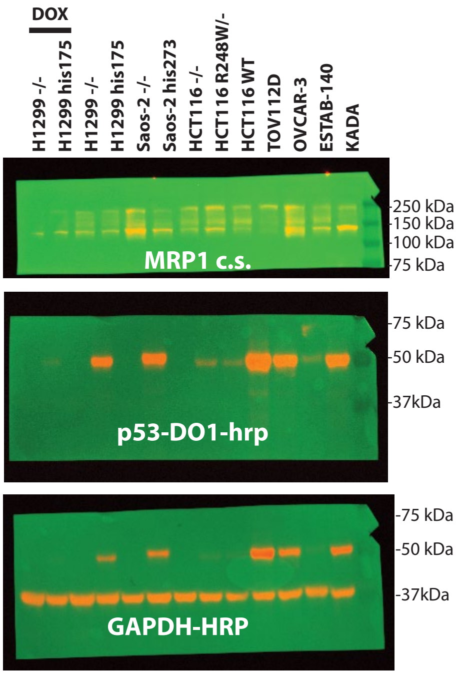

Supplement: Supplementary file 3 — Source Data for Expanded View and Appendix [file EMMM-13-e10852-s007.zip › Appendix Figure Source Data.zip!/Fig S5.zip!/Figure S1D and S5A.pdf]
