# Supplementary material for: A thiol‐bound drug reservoir enhances APR‐246‐induced mutant p53 tumor cell death
Source: EMBO Mol Med. 2020 Dec 14;13(2):e10852. doi: 10.15252/emmm.201910852 (PMC7863383; doi:10.15252/emmm.201910852)
Supplement: Supplementary file 3 — Source Data for Expanded View and Appendix [file EMMM-13-e10852-s007.zip › Appendix Figure Source Data.zip!/Fig S7.zip!/Figure S7A.pdf]

Fig. S7A

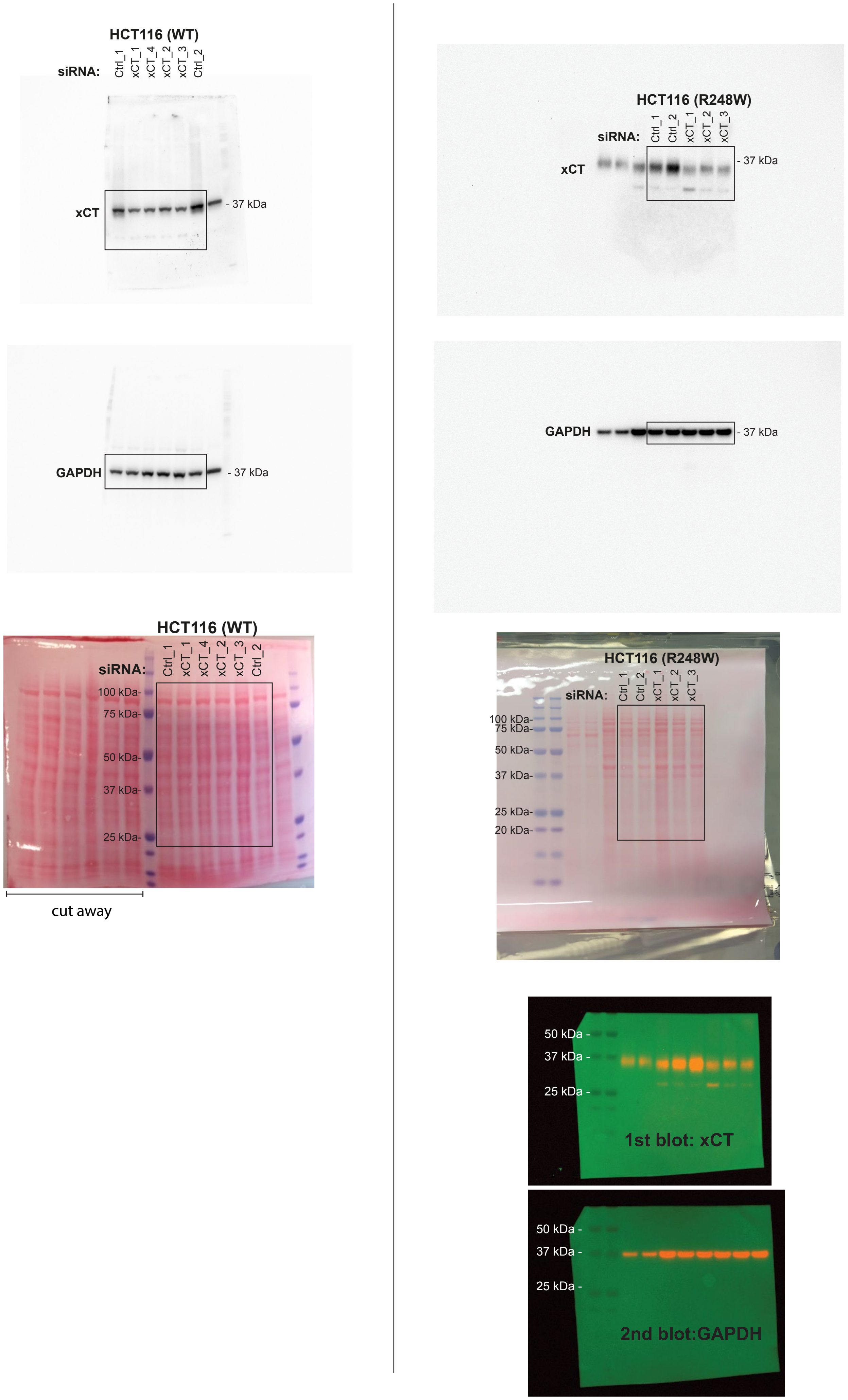
HCT116 (WT)
siRNA:
Ctrl_1
xCT_1
xCT_4
xCT_2
xCT_3
Ctrl_2
xCT
- 37 kDa
GAPDH
- 37 kDa
HCT116 (WT)
siRNA:
Ctrl_1
xCT_1
xCT_4
xCT_2
xCT_3
Ctrl_2
100 kDa-
75 kDa-
50 kDa-
37 kDa-
25 kDa-
cut away
HCT116 (R248W)
siRNA:
Ctrl_1
Ctrl_2
xCT_1
xCT_2
xCT_3
xCT
- 37 kDa
GAPDH
- 37 kDa
HCT116 (R248W)
siRNA:
Ctrl_1
Ctrl_2
xCT_1
xCT_2
xCT_3
100 kDa-
75 kDa-
50 kDa-
37 kDa-
25 kDa-
20 kDa-
50 kDa -
37 kDa -
25 kDa -
1st blot: xCT
50 kDa -
37 kDa -
25 kDa -
2nd blot:GAPDH
