# Supplementary material for: A thiol‐bound drug reservoir enhances APR‐246‐induced mutant p53 tumor cell death
Source: EMBO Mol Med. 2020 Dec 14;13(2):e10852. doi: 10.15252/emmm.201910852 (PMC7863383; doi:10.15252/emmm.201910852)
Supplement: Supplementary file 3 — Source Data for Expanded View and Appendix [file EMMM-13-e10852-s007.zip › Appendix Figure Source Data.zip!/Fig S7.zip!/Figure S7D.pdf]

Fig. S7D

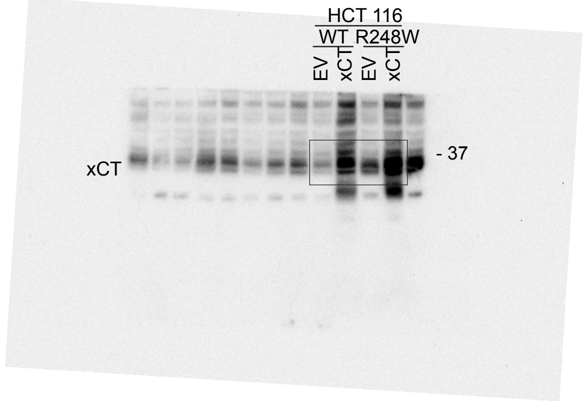

HCT 116
WT R248W
EV xCT EV xCT
xCT
- 37

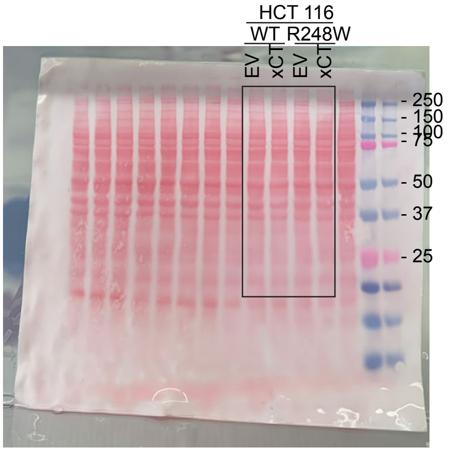

HCT 116
WT R248W
EV xCT EV xCT
- 250
- 150
- 100
- 75
- 50
- 37
- 25

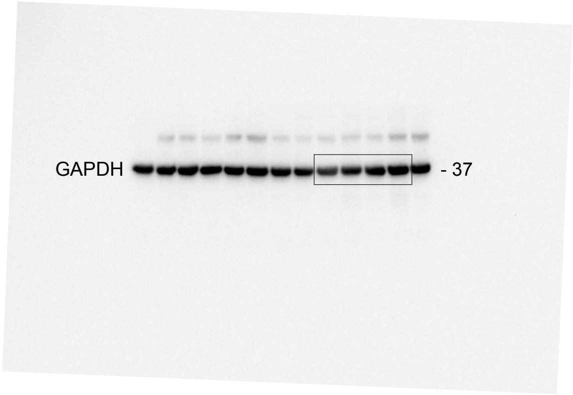

GAPDH
- 37

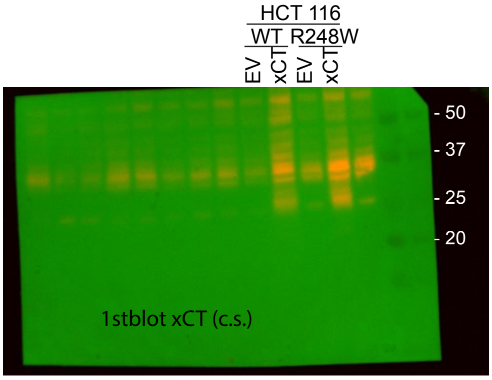

HCT 116
WT R248W
EV xCT EV xCT
- 50
- 37
- 25
- 20
1stblot xCT (c.s.)

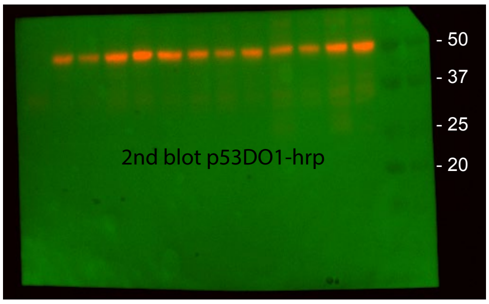

- 50
- 37
- 25
- 20
2nd blot p53DO1-hrp

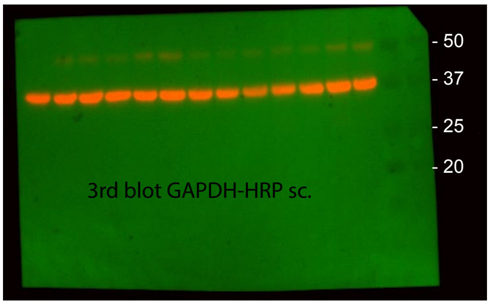

- 50
- 37
- 25
- 20
3rd blot GAPDH-HRP sc.
