# Supplementary material for: A thiol‐bound drug reservoir enhances APR‐246‐induced mutant p53 tumor cell death
Source: EMBO Mol Med. 2020 Dec 14;13(2):e10852. doi: 10.15252/emmm.201910852 (PMC7863383; doi:10.15252/emmm.201910852)
Supplement: Supplementary file 3 — Source Data for Expanded View and Appendix [file EMMM-13-e10852-s007.zip › Appendix Figure Source Data.zip!/Fig S6.zip!/Figure S6G.pdf]

Fig. S6G

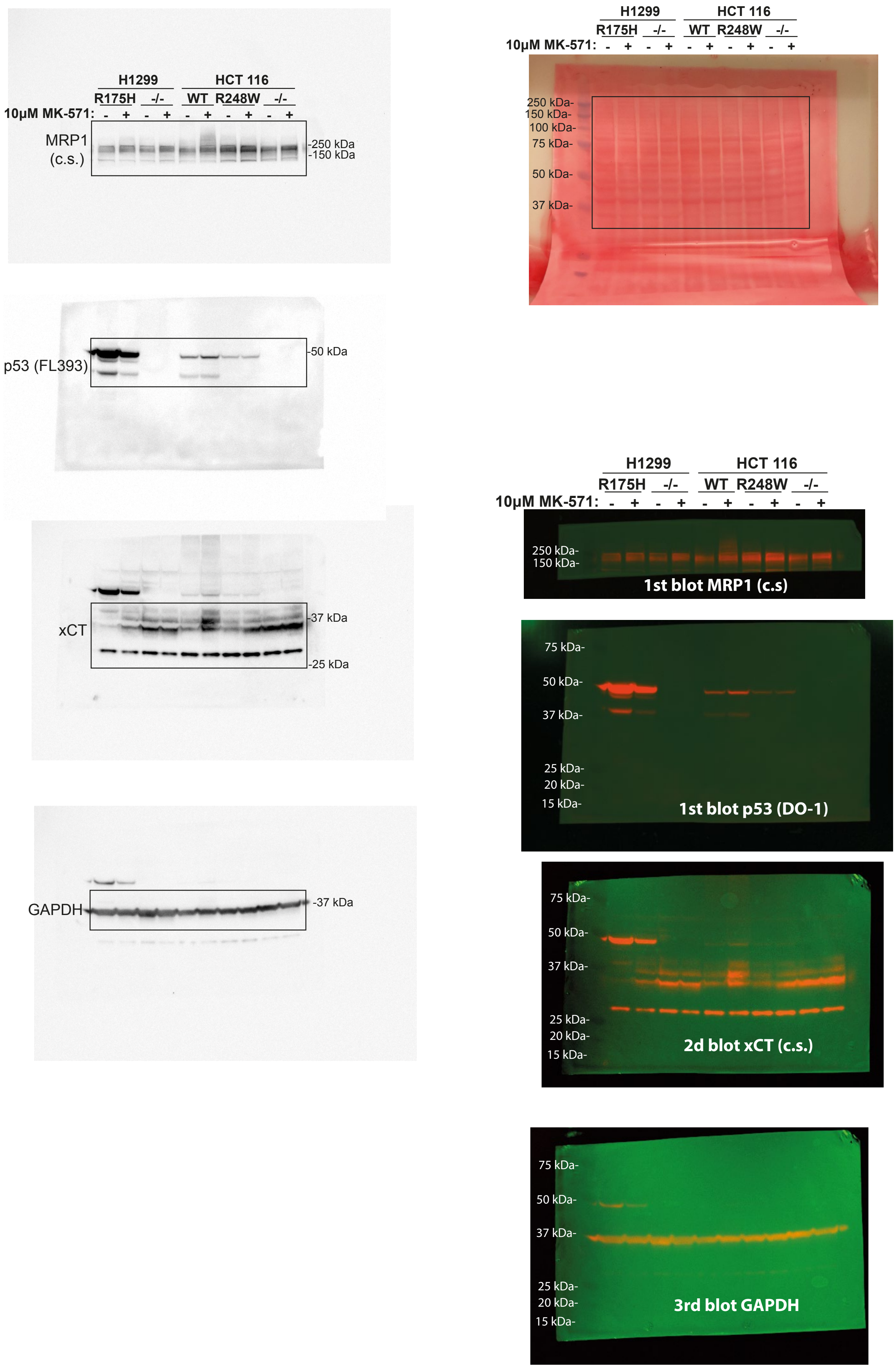
H1299
HCT 116
R175H
-/-
WT
R248W
-/-
10µM MK-571: - + - + - + - + - +
MRP1 (c.s.)
-250 kDa
-150 kDa
p53 (FL393)
-50 kDa
xCT
-37 kDa
-25 kDa
GAPDH
-37 kDa
250 kDa-
150 kDa-
100 kDa-
75 kDa-
50 kDa-
37 kDa-
1st blot MRP1 (c.s)
25 kDa-
20 kDa-
15 kDa-
1st blot p53 (DO-1)
2d blot xCT (c.s.)
3rd blot GAPDH
