# Supplementary material for: A thiol‐bound drug reservoir enhances APR‐246‐induced mutant p53 tumor cell death
Source: EMBO Mol Med. 2020 Dec 14;13(2):e10852. doi: 10.15252/emmm.201910852 (PMC7863383; doi:10.15252/emmm.201910852)
Supplement: Supplementary file 3 — Source Data for Expanded View and Appendix [file EMMM-13-e10852-s007.zip › Appendix Figure Source Data.zip!/Fig S6.zip!/Figure S6B.pdf]

Fig. S6B

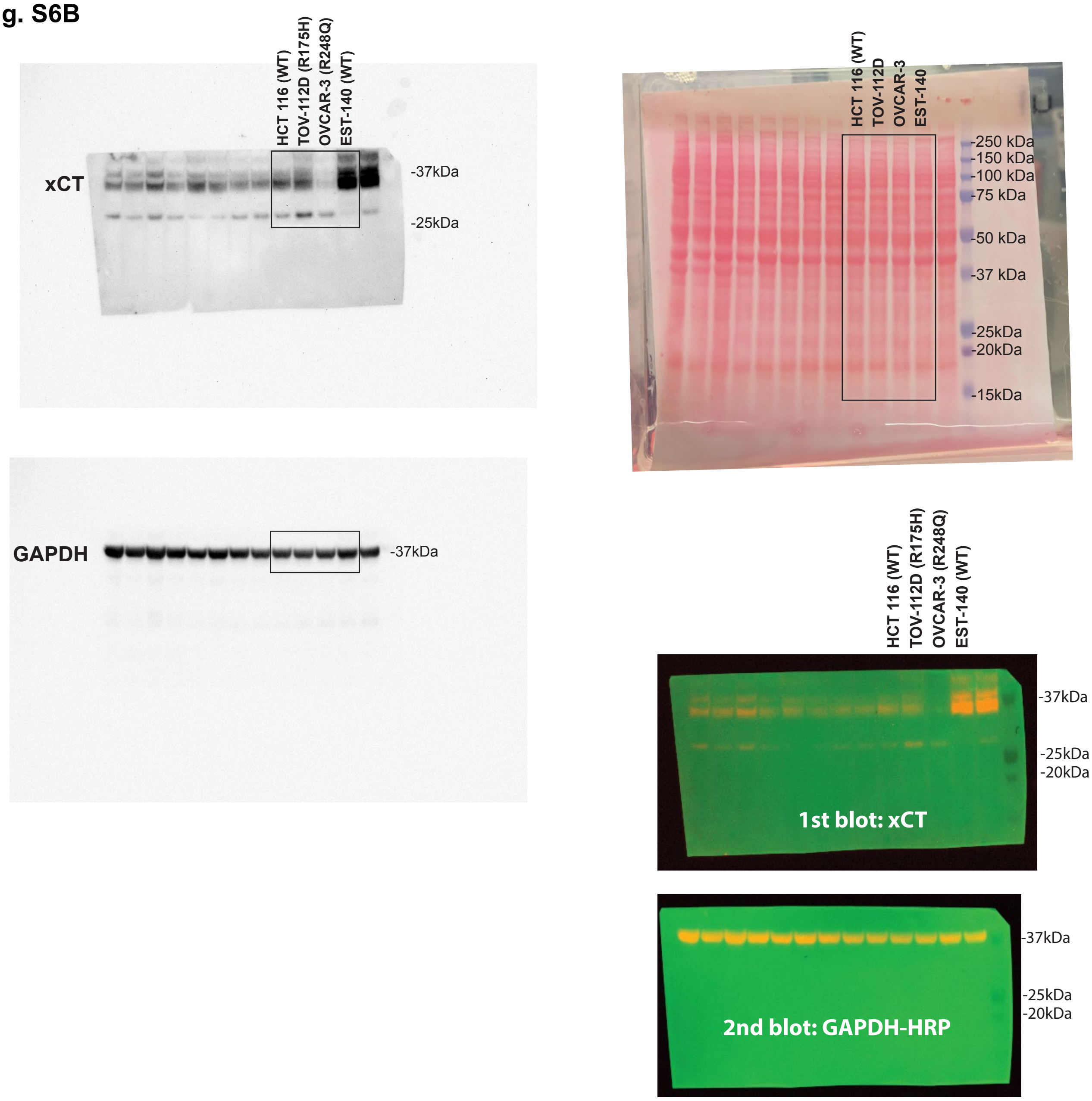
HCT 116 (WT)
TOV-112D (R175H)
OVCAR-3 (R248Q)
EST-140 (WT)
xCT
-37kDa
-25kDa
GAPDH
-37kDa
HCT 116 (WT)
TOV-112D
OVCAR-3
EST-140
-250 kDa
-150 kDa
-100 kDa
-75 kDa
-50 kDa
-37 kDa
-25kDa
-20kDa
-15kDa
HCT 116 (WT)
TOV-112D (R175H)
OVCAR-3 (R248Q)
EST-140 (WT)
-37kDa
-25kDa
-20kDa
1st blot: xCT
-37kDa
-25kDa
-20kDa
2nd blot: GAPDH-HRP
