# Supplementary material for: A thiol‐bound drug reservoir enhances APR‐246‐induced mutant p53 tumor cell death
Source: EMBO Mol Med. 2020 Dec 14;13(2):e10852. doi: 10.15252/emmm.201910852 (PMC7863383; doi:10.15252/emmm.201910852)
Supplement: Supplementary file 3 — Source Data for Expanded View and Appendix [file EMMM-13-e10852-s007.zip › Appendix Figure Source Data.zip!/Fig S1.zip!/Figure S1I.pdf]

**Fig. S1I**

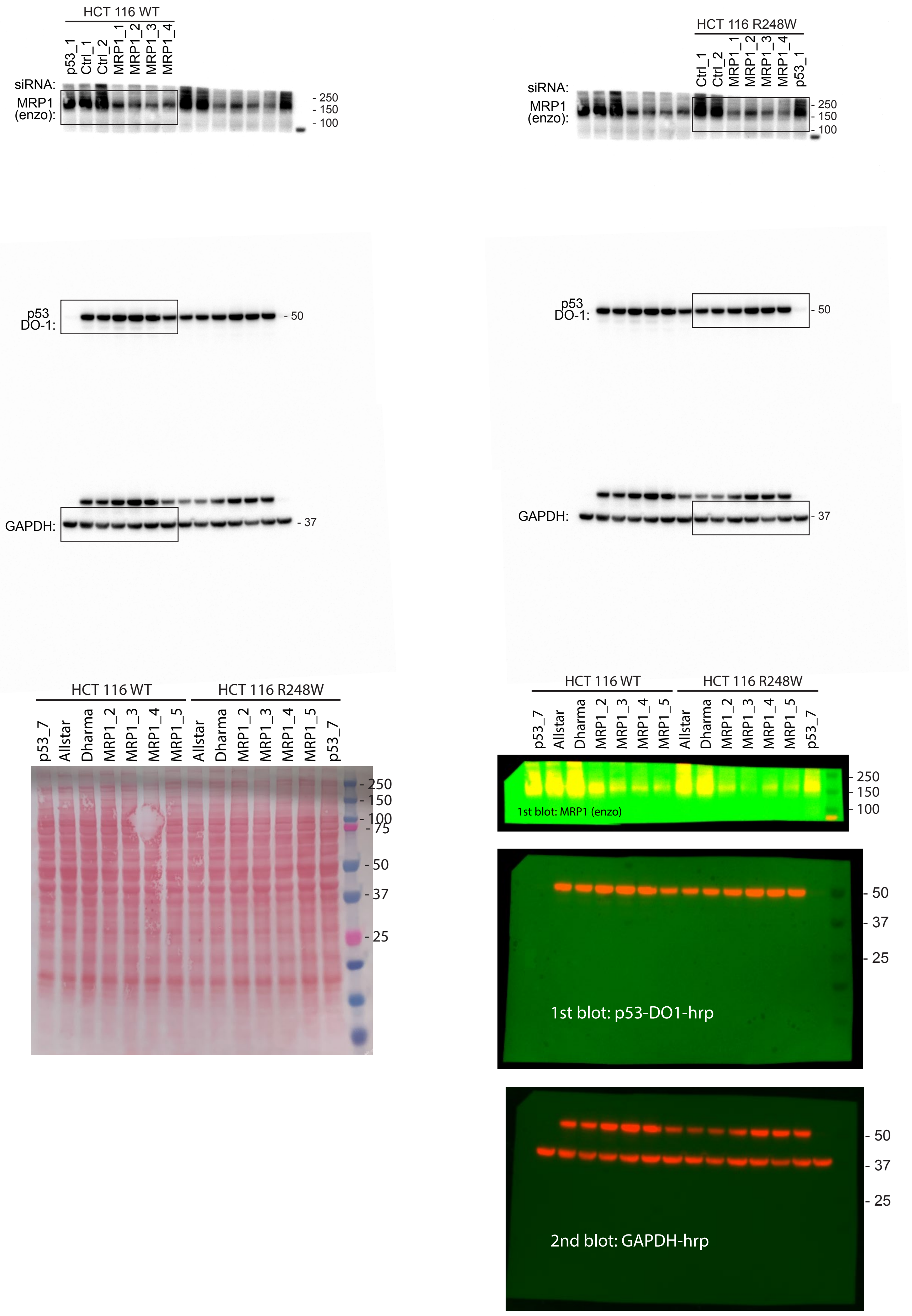
HCT 116 WT
p53_1
Ctrl_1
Ctrl_2
MRP1_1
MRP1_2
MRP1_3
MRP1_4
siRNA:
MRP1 (enzo):
- 250
- 150
- 100
HCT 116 R248W
Ctrl_1
Ctrl_2
MRP1_1
MRP1_2
MRP1_3
MRP1_4
p53_1
p53 DO-1:
- 50
GAPDH:
- 37
HCT 116 WT
HCT 116 R248W
p53_7
Allstar
Dharma
MRP1_2
MRP1_3
MRP1_4
MRP1_5
- 75
- 25
1st blot: MRP1 (enzo)
1st blot: p53-DO1-hrp
2nd blot: GAPDH-hrp
