# Supplementary material for: A thiol‐bound drug reservoir enhances APR‐246‐induced mutant p53 tumor cell death
Source: EMBO Mol Med. 2020 Dec 14;13(2):e10852. doi: 10.15252/emmm.201910852 (PMC7863383; doi:10.15252/emmm.201910852)

Fig. S1E

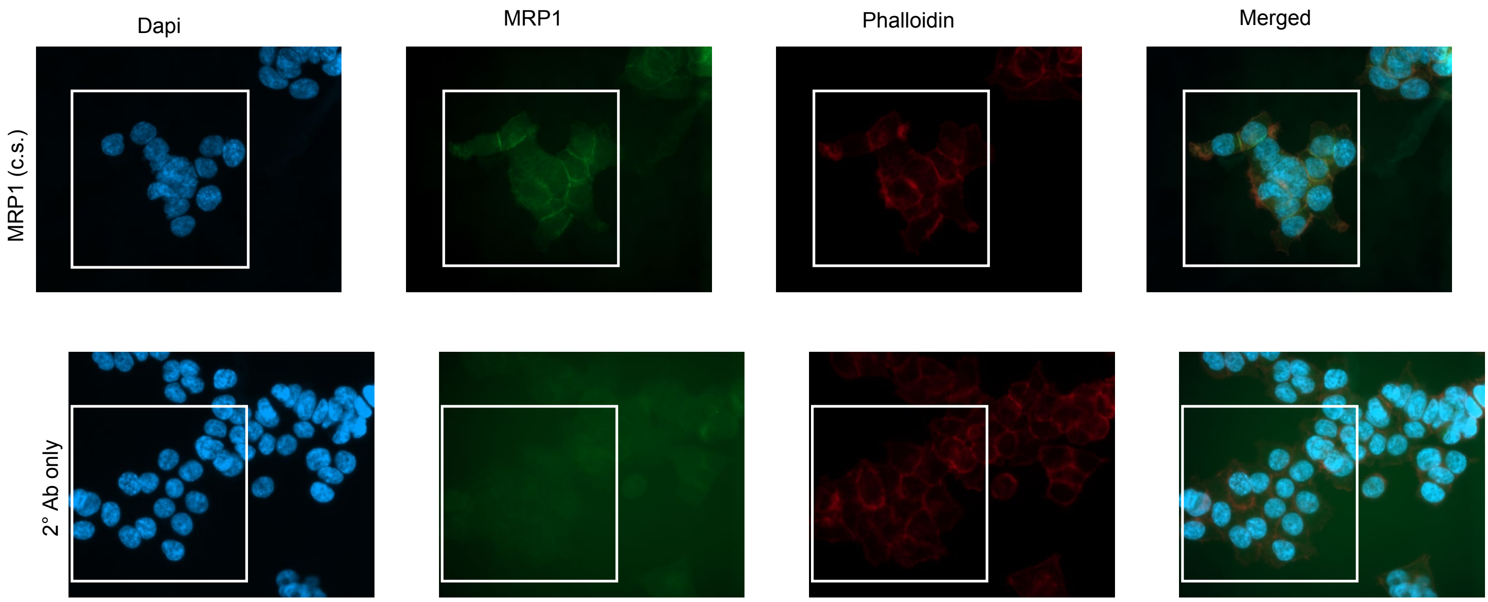

Supplement: Supplementary file 3 — Source Data for Expanded View and Appendix [file EMMM-13-e10852-s007.zip › Appendix Figure Source Data.zip!/Fig S1.zip!/Figure S1E.pdf]
